# Supplementary material for: Identical twins carry a persistent epigenetic signature of early genome programming
Source: Nat Commun. 2021 Sep 28;12:5618. doi: 10.1038/s41467-021-25583-7 (PMC8479069; doi:10.1038/s41467-021-25583-7)
Supplement: Supplementary file 1 — Supplementary Information [file 41467_2021_25583_MOESM1_ESM.pdf]

Supplementary Information

Supplementary Note 1 BIOS consortium members

Supplementary Note 2 Genetics of DNA Methylation Consortium Members

Supplementary Note 3 Results of Sensitivity analyses

Supplementary Note 4 Correlation between MZ-DMPs

Supplementary Note 5 Enrichment analyses EWAS atlas

Supplementary Note 6 Methylation QTL analyses

Supplementary Note 7 DNA methylation predictor of MZ twinning

Supplementary Note 8 Acknowledgements

Supplementary Figures

Supplementary Tables

Supplementary References

Supplementary Note 1 BIOS consortium members

BIOS Consortium (Biobank-based Integrative Omics Study)

Management Team Bastiaan T. Heijmans (chair)[1], Peter A.C. ’t Hoen[2], Joyce van Meurs[3], Aaron Isaacs[4], Rick Jansen[5], Lude Franke[6].

Cohort collection Dorret I. Boomsma[7], René Pool[7], Jenny van Dongen[7], Jouke J. Hottenga[7] (Netherlands Twin Register); Marleen MJ van Greevenbroek[8], Coen D.A. Stehouwer[8], Carla J.H. van der Kallen[8], Casper G. Schalkwijk[8] (Cohort study on Diabetes and Atherosclerosis Maastricht); Cisca Wijmenga[6], Lude Franke[6], Sasha Zhernakova[6], Ettje F. Tigchelaar[6] (LifeLines Deep); P. Eline Slagboom[1], Marian Beekman[1], Joris Deelen[1], Diana van Heemst[9] (Leiden Longevity Study); Jan H. Veldink[10], Leonard H. van den Berg[10] (Prospective ALS Study Netherlands); Cornelia M. van Duijn[4], Bert A. Hofman[11], Aaron Isaacs[4], André G. Uitterlinden[3] (Rotterdam Study).

Data Generation Joyce van Meurs (Chair)[3], P. Mila Jhamai[3], Michael Verbiest[3], H. Eka D. Suchiman[1], Marijn Verkerk[3], Ruud van der Breggen[1], Jeroen van Rooij[3], Nico Lakenberg[1].

Data management and computational infrastructure Hailiang Mei (Chair)[12], Maarten van Iterson[1], Michiel van Galen[2], Jan Bot[13], Dasha V. Zhernakova[6], Rick Jansen[5], Peter van ’t Hof[12], Patrick Deelen[6], Irene Nooren[13], Peter A.C. ’t Hoen[2], Bastiaan T. Heijmans[1], Matthijs Moed[1].

Data Analysis Group Lude Franke (Co-Chair)[6], Martijn Vermaat[2], Dasha V. Zhernakova[6], René Luijk[1], Marc Jan Bonder[6], Maarten van Iterson[1], Patrick Deelen[6], Freerk van Dijk[14], Michiel van Galen[2], Wibowo Arindrarto[12], Szymon M. Kielbasa[15], Morris A. Swertz[14], Erik. W van Zwet[15], Rick Jansen[5], Peter-Bram ’t Hoen (Co-Chair)[2], Bastiaan T. Heijmans (Co-Chair)[1].

1. Molecular Epidemiology Section, Department of Medical Statistics and Bioinformatics, Leiden University Medical Center, Leiden, The Netherlands
2. Department of Human Genetics, Leiden University Medical Center, Leiden, The Netherlands
3. Department of Internal Medicine, ErasmusMC, Rotterdam, The Netherlands
4. Department of Genetic Epidemiology, ErasmusMC, Rotterdam, The Netherlands
5. Department of Psychiatry, VU University Medical Center, Neuroscience Campus Amsterdam, Amsterdam, The Netherlands
6. Department of Genetics, University of Groningen, University Medical Centre Groningen, Groningen, The Netherlands
7. Department of Biological Psychology, VU University Amsterdam, Neuroscience Campus Amsterdam, Amsterdam, The Netherlands
8. Department of Internal Medicine and School for Cardiovascular Diseases (CARIM), Maastricht University Medical Center, Maastricht, The Netherlands
9. Department of Gerontology and Geriatrics, Leiden University Medical Center, Leiden, The Netherlands
10. Department of Neurology, Brain Center Rudolf Magnus, University Medical Center Utrecht, Utrecht, The Netherlands
11. Department of Epidemiology, ErasmusMC, Rotterdam, The Netherlands
12. Sequence Analysis Support Core, Leiden University Medical Center, Leiden, The Netherlands
13. SURFsara, Amsterdam, the Netherlands
14. Genomics Coordination Center, University Medical Center Groningen, University of Groningen, Groningen, the Netherlands
15. Medical Statistics Section, Department of Medical Statistics and Bioinformatics, Leiden University Medical Center, Leiden, The Netherlands

Supplementary Note 2 Genetics of DNA Methylation Consortium members

Josine L Min[1,2*], Gibran Hemani[1,2*], Eilis Hannon[3], Koen F Dekkers[4], Juan Castillo-Fernandez[5], René Luijk[4], Elena Carnero-Montoro[5,6], Daniel J Lawson[1,2], Kimberley Burrows[1,2], Matthew Suderman[1,2], Andrew D Bretherick[7], Tom G Richardson[1,2], Johanna Klughammer[8], Valentina Iotchkova[9], Gemma Sharp[1,2], Ahmad Al Khleifat[10], Aleksey Shatunov[10], Alfredo Iacoangeli[10,11], Wendy L McArdle[2], Karen M Ho[2], Ashish Kumar[12,13,14], Cilla Söderhäll[15], Carolina Soriano-Tárraga[16], Eva Giralt-Steinhauer[16], Nabila Kazmi[1,2], Dan Mason[17], Allan F McRae[18], David L Corcoran[19], Karen Sugden[19,20], Silva Kasela[21], Alexia Cardona[22,23], Felix R Day[22], Giovanni Cugliari[24,25], Clara Viberti[24,25], Simonetta Guarrera[24,25], Michael Lerro[26], Richa Gupta[27,28], Sailalitha Bollepalli[27,28], Pooja Mandaviya[29], Yanni Zeng[7,30,31], Toni-Kim Clarke[32], Rosie M Walker[33,34], Vanessa Schmoll[35], Darina Czamara[35], Carlos Ruiz-Arenas[36,37,38], Faisal I Rezwan[39], Riccardo E Marioni[33,34], Tian Lin[18], Yvonne Awaloff[35], Marine Germain[40], Dylan Aïssi[41], Ramona Zwamborn[42], Kristel van Eijk[42], Annelot Dekker[42], Jenny van Dongen[43], Jouke-Jan Hottenga[43], Gonneke Willemsen[43], Cheng-Jian Xu[44,45], Guillermo Barturen[6], Francesc Català-Moll[46], Martin Kerick[47], Carol Wang[48], Phillip Melton[49], Hannah R Elliott[1,2], Jean Shin[50], Manon Bernard[50], Idil Yet[5,51], Melissa Smart[52], Tyler Gorrie-Stone[53], BIOS Consortium[54], Chris Shaw[10,55], Ammar Al Chalabi[10,55,56], Susan M Ring[1,2], Göran Pershagen[12], Erik Melén[12,57], Jordi Jiménez-Conde[16], Jaume Roquer[16], Deborah A Lawlor[1,2], John Wright[17], Nicholas G Martin[58], Grant W Montgomery[18], Terrie E Moffitt[19,20,59,62], Richie Poulton[60], Tõnu Esko[21,61], Lili Milani[21], Andres Metspalu[21], John RB Perry[22], Ken K Ong[22], Nicholas J Wareham[22], Giuseppe Matullo[24,25], Carlotta Sacerdote[25,63], Salvatore Panico[64], Avshalom Caspi[19,20,59,62], Louise Arseneault[62], France Gagnon[26], Miina Ollikainen[27,28], Jaakko Kaprio[27,28], Janine F Felix[65,66], Fernando Rivadeneira[29], Henning Tiemeier[67,68], Marinus H van IJzendoorn[69,70], André G Uitterlinden[29], Vincent WV Jaddoe[65,66], Chris Haley[7], Andrew M McIntosh[32,34], Kathryn L Evans[33,34], Alison Murray[71], Katri Räikkönen[72], Jari Lahti[72], Ellen A Nohr[73,74], Thorkild IA Sørensen[1,2,75,76], Torben Hansen[75], Camilla S Morgen[75,77], Elisabeth B Binder[35,78], Susanne Lucae[35], Juan Ramon Gonzalez[36,37,38], Mariona Bustamante[36,37,38,79], Jordi Sunyer[36,37,38,80], John W Holloway[81,82], Wilfried Karmaus[83], Hongmei Zhang[83], Ian J Deary[34], Naomi R Wray[18,84], John M Starr[34,85], Marian Beekman[4], Diana van Heemst[86], P Eline Slagboom[4], Pierre-Emmanuel Morange[87], David-Alexandre Trégouët[40], Jan H Veldink[42], Gareth E Davies[88], Eco JC de Geus[43], Dorret I Boomsma[43], Judith M Vonk[89], Bert Brunekreef[90,91], Gerard H Koppelman[44], Marta E Alarcón-Riquelme[6,12], Rae-Chi Huang[92], Craig E Pennell[48], Joyce van Meurs[29], M Arfan Ikram[93], Alun D Hughes[94], Therese Tillin[94], Nish Chaturvedi[94], Zdenka Pausova[50], Tomas Paus[95], Timothy D Spector[5], Meena Kumari[52], Leonard C Schalkwyk[53], Peter M Visscher[18,84], George Davey Smith[1,2], Christoph Bock[8,96], Tom R Gaunt[1,2], Jordana T Bell[5‡], Bastiaan T Heijmans[4‡], Jonathan Mill[3‡], Caroline L Relton[1,2‡]

* These authors contributed equally to this research.
[‡]These authors jointly supervised this work.
Corresponding author: Josine L Min, josine.min@bristol.ac.uk

Affiliations

[1] MRC Integrative Epidemiology Unit, University of Bristol, Bristol, UK
[2] Population Health Sciences, Bristol Medical School, University of Bristol, Bristol, UK
[3] University of Exeter Medical School, College of Medicine and Health, University of Exeter, Exeter, UK

[4] Molecular Epidemiology, Department of Biomedical Data Sciences, Leiden University Medical Center, Leiden, The Netherlands
[5] Department of Twin Research and Genetic Epidemiology, King's College London, London, UK
[6] Pfizer - University of Granada - Andalusian Government Center for Genomics and Oncological Research (GENYO), Spain
[7] MRC Human Genetics Unit, Institute of Genetics and Molecular Medicine, University of Edinburgh, Edinburgh, UK
[8] CeMM Research Center for Molecular Medicine of the Austrian Academy of Sciences, Vienna, Austria
[9] MRC Weatherall Institute of Molecular Medicine, Oxford, UK
[10] Department of Basic and Clinical Neuroscience, Maurice Wohl Clinical Neuroscience Institute, London, UK
[11] Department of Biostatistics and Health Informatics, King's College London, London, UK
[12] Institute of Environmental Medicine, Karolinska Institutet, Stockholm, Solna, Sweden
[13] Chronic Disease Epidemiology unit, Swiss Tropical and Public Health Institute, Basel, Switzerland
[14] University of Basel, Basel, Switzerland
[15] Department of Women's and Children's Health, Karolinska Institutet, Stockholm, Sweden
[16] Neurology Department, Hospital del Mar - IMIM (Institut Hospital del Mar d'Investigacions Mèdiques), Barcelona, Spain
[17] Bradford Institute for Health Research, Bradford, UK
[18] Institute for Molecular Bioscience, University of Queensland, Australia
[19] Center for Genomic and Computational Biology, Duke University, Durham, NC, USA
[20] Department of Psychology and Neuroscience, Duke University, Durham, NC, USA
[21] Estonian Genome Center, Institute of Genomics, University of Tartu, Estonia
[22] MRC Epidemiology Unit, University of Cambridge, School of Clinical Medicine, Institute of Metabolic Science, Cambridge Biomedical Campus, Cambridge CB2 0QQ, United Kingdom
[23] Department of Genetics, University of Cambridge, Downing Street, Cambridge CB2 3EH, United Kingdom
[24] Department of Medical Sciences, University of Turin, Turin, Italy
[25] Italian Institute for Genomic Medicine (IIGM), Turin, Italy
[26] Dalla Lana School of Public Health, University of Toronto, Toronto, Canada
[27] Institute for Molecular Medicine, University of Helsinki, Helsinki, Finland
[28] Department of Public Health, Faculty of Medicine, University of Helsinki, Helsinki, Finland
[29] Department of Internal Medicine, Erasmus University Medical Center, Rotterdam, The Netherlands
[30] Faculty of Forensic Medicine, Zhongshan School of Medicine, Sun Yat-Sen University, Guangzhou, China
[31] Guangdong Province Key Laboratory of Brain Function and Disease, Zhongshan School of Medicine, Sun Yat-Sen University, Guangzhou, China
[32] Division of Psychiatry, Royal Edinburgh Hospital, University of Edinburgh, Edinburgh EH10 5HF, UK
[33] Centre for Genomic and Experimental Medicine, Institute of Genetics and Molecular Medicine, Western General Hospital, University of Edinburgh, Crewe Road, Edinburgh EH4 2XU, UK
[34] Centre for Cognitive Ageing and Cognitive Epidemiology, Department of Psychology, University of Edinburgh, 7 George Square, Edinburgh EH8 9JZ, UK
[35] Department of Translational Research in Psychiatry, Max-Planck-Institute of Psychiatry, Munich, Germany
[36] ISGlobal, Barcelona Global Health Institute, Barcelona, Spain
[37] Universitat Pompeu Fabra (UPF), Barcelona, Spain
[38] CIBER Epidemiología y Salud Pública (CIBERESP), Madrid, Spain
[39] School of Water, Energy and Environment, Cranfield University, Cranfield, Bedfordshire MK43 0AL, UK

[40] INSERM UMR_S 1219, Bordeaux Population Health Center, University of Bordeaux, 33076 Bordeaux Cedex, France
[41] Department of General and Interventional Cardiology, University Heart Center Hamburg, Hamburg, Germany
[42] Department of Neurology, Brain Center Rudolf Magnus, University Medical Center Utrecht, Utrecht, 3584 CG, The Netherlands
[43] Department of Biological Psychology, Amsterdam Public Health Research Institute, Vrije Universiteit Amsterdam, Van Der Boechorststraat 7-9, 1081 BT, Amsterdam, The Netherlands
[44] University of Groningen, University Medical Center Groningen, Department of Pediatric Pulmonology and Pediatric Allergology, Beatrix Children's Hospital, GRIAC Research Institute Groningen, The Netherlands
[45] CiiM and TWINCORE, joint ventures between the Hannover Medical School and the Helmholtz Centre for Infection Research, Hannover, Germany
[46] Chromatin and Disease Group, Cancer Epigenetics and Biology Programme (PEBC), Bellvitge Biomedical Research Institute (IDIBELL), 08908 L'Hospitalet de Llobregat, Barcelona, Spain
[47] Instituto de Parasitología y Biomedicina López Neyra, CSIC, Granada, Spain
[48] School of Medicine and Public Health, College of Health, Medicine and Wellbeing, The University of Newcastle, Newcastle, NSW, Australia
[49] Menzies Institute for Medical Research, College of Health and Medicine, University of Tasmania, Hobart, Australia, School of Global Population Health, Faculty of Health and Medical Sciences, The University of Western Australia, Perth, WA, Australia; School of Pharmacy and Biomedical Sciences, Faculty of Health Sciences, Curtin University, Perth, WA, Australia
[50] The Hospital for Sick Children, University of Toronto, Toronto, Canada
[51] Department of Bioinformatics, Institute of Health Sciences, Hacettepe University, 06100, Ankara, Turkey
[52] Institute for Social and Economic Research, University of Essex, Wivenhoe Park, Colchester, Essex, CO4 3SQ
[53] School of Life Sciences, University of Essex, Wivenhoe Park, Colchester, Essex, CO4 3SQ
[54] A list of consortium authors and affiliations appears at the end of the paper.
[55] Department of Neurology, King's College Hospital, London, UK
[56] United Kingdom Dementia Research Institute, King's College London, London, UK
[57] Department of Clinical Science and Education, Södersjukhuset, Karolinska Institutet, Stockholm, Sweden
[58] QIMR Berghofer Medical Research Institute, Brisbane, Australia
[59] Department of Psychiatry and Behavioral Sciences, Duke University Medical School, Durham, NC, USA
[60] Dunedin Multidisciplinary Health and Development Research Unit, Department of Psychology, University of Otago, Dunedin, New Zealand
[61] Program in Medical and Population Genetics, Broad Institute, Broad Institute, Cambridge, MA, USA
[62] Social, Genetic and Developmental Psychiatry Centre, Institute of Psychiatry, Psychology and Neuroscience, King's College London, London, UK
[63] Piemonte Centre for Cancer Prevention, Turin, Italy
[64] Dipartimento Di Medicina Clinica E Chirurgia, Federico II University, Naples, Italy
[65] The Generation R Study Group, Erasmus MC, University Medical Center Rotterdam, Rotterdam, The Netherlands
[66] Department of Pediatrics, Erasmus MC, University Medical Center Rotterdam, Rotterdam, The Netherlands
[67] Department of Child and Adolescent Psychiatry, Erasmus Medical Center, Rotterdam, Netherlands

[68] Department of Social and Behavioral Science, Harvard TH Chan School of Public Health, Boston, USA
[69] Department of Psychology, Education and Child Studies, Erasmus University Rotterdam, The Netherlands
[70] Department of Clinical, Educational and Health Psychology, Division on Psychology and Language Sciences, Faculty of Brain Sciences, UCL, London, UK
[71] Institute of Medical Sciences, University of Aberdeen, Aberdeen, UK
[72] Department of Psychology and Logopedics, Faculty of Medicine, University of Helsinki, Finland
[73] Research Unit for Gynaecology and Obstetrics, Institute of Clinical research, University of Southern Denmark, Odense, Denmark
[74] Centre of Women's, Family and Child Health, University of South-Eastern Norway, Kongsberg, Norway
[75] The Novo Nordisk Foundation Center for Basic Metabolic Research, Faculty of Health and Medical Sciences, University of Copenhagen, Denmark
[76] Department of Public Health (Section of Epidemiology), Faculty of Health and Medical Sciences, University of Copenhagen, Copenhagen, Denmark.
[77] The National Institute of Public Health, University of Southern Denmark, Copenhagen
[78] Department of Psychiatry and Behavioral Sciences, Emory University School of Medicine, Atlanta, GA, USA
[79] Center for Genomic Regulation (CRG), Barcelona Institute of Science and Technology, Barcelona, Spain
[80] IMIM (Hospital del Mar Medical Research Institute), Barcelona, Spain
[81] Human Development and Health, Faculty of Medicine, University of Southampton, Southampton, UK
[82] Clinical and Experimental Sciences, Faculty of Medicine, University of Southampton, Southampton, UK
[83] Division of Epidemiology, Biostatistics, and Environmental Health Sciences, School of Public Health, University of Memphis, Memphis, USA
[84] Queensland Brain Institute, University of Queensland, Australia
[85] Alzheimer Scotland Dementia Research Centre, University of Edinburgh, University of Edinburgh, UK
[86] Department of Gerontology and Geriatrics, Leiden University Medical Center, Leiden, The Netherlands
[87] C2VN, Aix-Marseille University, INSERM, INRAE, Marseille, France
[88] Avera Institute for Human Genetics, Sioux Falls, USA
[89] University of Groningen, University Medical Center Groningen, Department of Epidemiology, GRIAC Research Institute Groningen, Groningen, The Netherlands
[90] Institute for Risk Assessment Sciences, Universiteit Utrecht, Utrecht, The Netherlands
[91] Julius Center for Health Sciences and Primary Care, University Medical Center Utrecht, Utrecht, The Netherlands
[92] Telethon Kids Institute, University of Western Australia, Perth, WA, Australia
[93] Department of Epidemiology, Erasmus MC, University Medical Center Rotterdam, Rotterdam, The Netherlands
[94] UCL Institute of Cardiovascular Science, London, UK
[95] Departments of Psychology and Psychiatry, University of Toronto, Toronto, Canada
[96] Institute of Artificial Intelligence and Decision Support, Center for Medical Statistics, Informatics, and Intelligent Systems, Medical University of Vienna, Vienna, Austria

Supplementary Note 3 Results of Sensitivity analyses

Parents and siblings as control group

In total, 24 sites were Bonferroni significant in the EWAS (N=1161) with MZ twins as cases, non-twins, i.e. parents and siblings, as controls (and all DZ twins excluded).

The effect sizes of the 243 epigenome-wide significant sites detected in NTR with the primary analysis reported in this paper (MZ twins versus DZ twins, N=1957) correlated strongly with effect sizes obtained by comparing MZ twins to parents and siblings and 242 sites (99.6%) showed the same direction of effect (r=0.96, p< 2.2e-16, **Figure S1A, Supplementary Data 2**).

By contrast, in an EWAS of DZ twins versus non-twins (N= 1270, all MZ twins were excluded), 0 sites were Bonferroni significant, the correlation between effect sizes was -0.34 (p-value = 3.6×10^{-8} , **figure S1B, Supplementary Data 1**) and 90 showed the same direction of effect as in the comparison of DZ versus MZ twins. These results indicate that the results from our primary EWAS (mainly) reflect differential DNA methylation in MZ twins.

In the Brisbane System Genetics Study (BSGS), which 125 MZ twins, 194 DZ twins, 95 siblings of twins, and 62 parents of twins, the 243 CpGs detected in NTR showed equally strong concordance of effects when comparing MZ twins to DZ twins as when comparing MZ twins to everyone else (DZ twins, siblings and parents; r=0.91, p< 2.2e-16, **Figure S2, Supplementary Data 1**).

Complete twin pairs versus single twins

We compared the primary EWAS approach with one randomly excluded MZ twin for each pair to 1) an EWAS performed in gee with complete MZ pairs and complete DZ pairs included and 2) an EWAS with a simple linear model (R function lm()) with only one randomly selected twin of each MZ and one randomly selected twin of each DZ pair included.

In total, 243 sites were epigenome-wide significant in the primary analysis reported in this paper (N=1957), 258 sites were epigenome-wide significant in the analysis that included all twins, including complete MZ twin pairs and complete DZ twin pairs, and twins from incomplete pairs (N=2750), and 130 sites were epigenome-wide significant in the analysis of single MZ twins and single DZ twins (N=1538).

The effect sizes of the 243 epigenome-wide significant sites detected in NTR with the primary analysis reported in this paper (single MZ twins and all DZ twins included, thus including complete DZ pairs and DZ twins from incomplete pairs) correlated strongly with effect sizes obtained with the analysis with all complete pairs included (r= 0.999**, figure S1C**)

and with the analysis with only single twins included (*r*= 0.996, **figure S1D**), and adding the extra MZ co-twin did not cause a large increase in the number of significant loci (this is expected if MZ twinning is associated with CpGs whose methylation levels are strongly correlated between MZ twins), with only 15 extra Bonferroni significant CpGs (6%).

Covariates

The primary EWAS analyses included the following covariates: age, sex, BMI, smoking, percentage of monocytes, percentage of eosinophils, percentage of neutrophils, sample plate and array row. We chose to correct for covariates that are known to be strongly associated with DNA methylation. In NTR, MZ and DZ twins showed small differences in the proportion of males and females, BMI, age, and sample plate, and no significant differences in cell counts.

In the analysis of single MZ twins and single DZ twins (N=1538) without any covariates included in the model, summary statistics showed inflation (lamba=1.35). After adjusting for inflation, 59 sites were epigenome-wide significant. The effect sizes of the 243 epigenome-wide significant sites detected in NTR with the primary analysis reported in this paper, with covariates age, sex, BMI, smoking, white blood cell percentages, sample plate and array row, correlated strongly with effect sizes obtained in the EWAS of single MZ twins and single DZ twins without any covariate (*r*= 0.99, **figure S1E**). All 243 DMPs showed the same direction of association without adjustment for any covariates.

Sex-stratified EWAS

In male twins and female twins separately, we performed an EWAS with a simple linear model (R function lm()) to compare female MZ twins to female DZ twins, and male MZ twins to male DZ twins. Only one randomly selected twin from each MZ pair and each DZ pair was included in these analyses.

In total, 28 sites were epigenome-wide significant in this analysis of female twins (N=1033) and 4 sites were epigenome-wide significant in the analysis of male twins (N=505).

The effect sizes of the 243 epigenome-wide significant sites detected in NTR with the primary analysis reported in this paper (single MZ twin and DZ pairs included) correlated strongly with effect sizes obtained in the analysis of females only (*r*= 0.99**, figure S1F**), and in the analysis of males only (*r*=0.97, **figure S1G**). Effect sizes obtained in the female EWAS also correlated strongly with the effect sizes obtained in the male EWAS (*r*=0.94, **figure S1H**).

Correction for top *cis* mQTL

We repeated the primary EWAS analysis in NTR for the DMPs detected in the meta-analysis adjusting, in addition to the same covariates as before (sex, age, cell counts, BMI, smoking, array row and sample plate), for genotype at the strongest *cis* mQTL SNP of each CpG (**Supplementary Note 6**) and three principal components (PCs) based on the genotype data. This analysis was performed in gee on all twins on which the primary EWAS analysis was performed (one randomly selected MZ twin and complete DZ twin pairs) for whom genotype data were available (N=1713). The analysis was conducted for 502 methylation sites with a significant *cis* mQTL and for which the SNP was available in NTR.

In total, 109 CpGs were associated with MZ versus DZ zygosity after adjusting for the strongest *cis* mQTL (plus the above-mentioned covariates) at $p < 1x10^{-7}$, and the effect size of zygosity was unaffected adjusting for the top *cis* mQTL (**Figure S20A**). Effect sizes of zygosity and *cis* mQTLs did not correlate (**Figure S20B**). Based on results from the same model, 251 CpGs were associated with the selected top-SNP in NTR at $p < 1x10^{-7}$ (**Figure S20C**) confirming the *cis* mQTL effect in this sample. These results suggest that *cis* mQTLs and zygosity are independently associated with methylation level at these CpGs. Histograms of the effect sizes of zygosity and *cis* mQTLs (taken from the same model) illustrate that the methylation differences between MZ and DZ twins are on average about half the effect size associated with each effect allele of *cis* mQTLs (**Figure S20D** and **Figure S20F**).

Supplementary Note 4 Correlation between MZ-DMPs

We explored whether methylation differences occur across extended stretches of DNA, which may indicate underlying regulatory mechanisms, and computed the correlation between DNA methylation levels in data from NTR to examine the extent to which the 834 MZ-DMPs are independent. While the average correlation across all 834 MZ-DMPs was small (mean=0.09, range=-0.69-0.98), the correlation between DMPs within a window of 1 Mb around each DMP with the most significant p-value was moderate (mean=0.53, range=0.04-0.98; for 99 windows containing 3 or more DMPs and an average size of 366 kb, range=7bp-1.4Mb). Examples of large regions are shown in **Fig. S3**.

We note that correlations between methylation levels at different CpGs may also arise due to cross-hybridization of probes to multiple locations. We note that we already excluded probes reported by Chen *et al* with an overlap of at least 47 bases per probe[1], which is the most commonly used exclusion criterium in EWA studies, from all of our analyses to avoid this issue. We additionally examined a more stringent definition based on a lower degree of sequence overlap of 30 bases per probe[2], which flagged 18 of the 834 MZ-DMPs (2.1%) as potentially cross-hybridizing. We have flagged these DMPs in **Supplementary Data 3.**

The sequence similarity of probes for the 834 MZ-DMPs was generally low. On average, 3.5 bases overlapped, the maximum overlap was 26 bases, and 685 CpGs (82%) are targeted by probes that show less than 14 bases overlap with probes for other MZ-DMPs. We examined one region in more detail; the *PCDH* gene clusters on chromosome 5, because the genes in this region are known to show large sequence similarity. Our EWAS meta-analysis identified 79 MZ-DMPs in this region (**Fig. S3B**). Among the 79 CpGs, the overlap in probe sequences between probes for different CpGs was on average only 3.5 bases, the maximum overlap was 21 bases and 77 of the 79 CpGs had less than 14 overlapping bases. This illustrates that the probes for these 79 CpGs are designed to target largely distinct sequences within the *PCDH* gene clusters, however, we note that all 79 CpGs are targeted by probes that show a small degree of off-target sequence overlap (>=14 bases[3]) with other sequences within this genomic region.

Supplementary Note 5 Enrichment analyses EWAS atlas

We performed enrichment analyses against all previously reported associations in EWASs of diseases and environmental exposures. The strongest enrichment for hypermethylated DMPs was folic acid supplementation during pregnancy (OR=293, $P=7.3x10^{-154}$), followed by neurodevelopmental presentations and congenital anomalies (OR= 65, $P=1.2x10^{-50}$), and Immunodeficiency, Centromeric instability, Facial anomalies syndrome (ICF syndrome; OR=174, $P=3.8x10^{-37}$), a rare disorder often caused by mutations in one of the DNA methyltransferase genes (*DNMT3B*). The strongest enrichment for hypomethylated CpGs was Kabuki Syndrome (OR=70, $P=9.5x10^{-159}$), a rare disorder caused by mutations in *KMT2D,* which codes for a histone lysine methyltransferase, and *KDM6A*, which codes for a histone lysine demethylase. Further enrichment was seen for a whole range of traits and exposures, including prenatal exposures, congenital anomalies, and preterm birth (**Supplementary Data 6 and 7)**. The enrichments further confirm that the MZ twinning epigenetic signature is linked to early-life epigenetic reprogramming.

Supplementary Note 6 Methylation QTL analyses

We obtained methylation QTL (mQTL) results for the 834 DMPs (497 hypomethylated, and 337 hypermethylated; **Supplementary Data 10 and 11**) from our EWAS in the largest mQTL catalogue to date; the whole blood mQTL results from the Genetics of DNA Methylation Consortium (GoDMC, N= 27,750)[4]. This revealed 108,241 significant *cis* associations between 365 hypo-DMPs and 61,823 genetic variants and 77,988 significant *cis* associations between 196 hyper-DMPs and 35,899 variants. In addition, there were 8,197 significant *trans* associations between 4,166 variants and 73 hypo-DMPs and 2,890 significant *trans* associations between 2,116 variants and 52 hyper-DMPs. *Trans* mQTLs were associated with up to 15 CpGs (average=1.8). Among the genes annotated to *trans* mQTLs were key epigenetic modifiers including *TRIM28 (trans* mQTL for hypomethylated DMPs) and the *de novo* methyltransferase *DNMT3B (trans* mQTL for hypomethylated DMPs), and a large number of zinc finger genes (for both hypomethylated and hypermethylated DMPs). SNPs with the largest number of *trans* effects were annotated to the *ZNF* gene cluster on chromosome 19 (up to 15 CpGs), and *DPPA4*[5,6], which encodes a key regulator of developmental pluripotency that interacts with the Polycomb Repressor Complex[7] (SNPs rs1044266, rs1163441, and rs2930074, each associated with 11 CpGs in *trans*). Dppa4 forms a heterodimer with Dppa2[8]. In line with the enrichment of hypomethylated DMPs within polycomb-repressed chromatin states, *DPPA2* and *DPPA4* are *trans* mQTLs for hypomethylated DMPs.

In line with the previously reported influence of genetic variants on MEs[9], 53 (77%) of the putative MEs associated with zygosity were associated with at least one mQTL *in cis*, and 14 (19%) were associated with at least one mQTL *in trans*.

We note that hypermethylated CpGs were significantly enriched in regions containing repeats; the effect of such repeats on DNA methylation is not fully captured in mQTL analyses. Thus, we cannot rule out that repeat variation might contribute to this DNA methylation signature.

In NTR, we examined whether association with zygosity remained after adjusting for the strongest *cis* mQTL at each site; results remained unchanged (**Supplementary Note 3, Figure S20**), illustrating that zygosity and *cis* mQTLs are independently associated with these methylation sites.

Supplementary Note 7 DNA methylation predictor of MZ twinning

We compared models based on two input sets (genome-wide methylation sites versus meta-analysis DMPs), and trained on two phenotypes (MZ versus DZ twins, and MZ twins versus everyone else (including DZ twins and family members of twins). Regressions returned predictors based on 232-1867 methylation sites (**Supplementary Data 12**). In NTR test data from blood (which were left out of the training dataset), the area under the curve (AUC) ranged from 0.69 to 0.77, with up to 84% of MZ twins correctly classified, up to 57% of DZ twins correctly classified, and up to 63% of family members correctly classified as non-MZ. We tested prediction in two independent datasets (**Table 1**): BSGS (blood from MZ twins, DZ twins, and family members, 450k array) and NTR children (buccal from MZ and DZ twins, EPIC array). AUCs ranged from 0.67 to 0.80 in BSGS, and from 0.63 to 0.76 in buccal data from NTR children. The predictors performed best when trained on genome-wide significant CpGs from the meta-analysis (rather than genome-wide methylation data). Weights of these scores are provided in **Supplementary Data 13** and **Supplementary Data 14**.

In the group of NTR children with buccal methylation data and information on chorionicity available, we compared the performance of the predictor for MZ twins with different chorionicities. The performance was similar across chorionicities. For the predictor that performed best on data from buccal (trained to distinguish MZ versus DZ twins, on genome-wide significant CpGs), the percentage of correctly predicted MZ twins were: 76% for monochorionic monoamniotic twins, 72% for monochorionic diamniotic twins, and 75% for dichorionic twins.

Supplementary Note 8 Acknowledgements

NTR adults (discovery cohort)

NTR warmly thanks all participants. We acknowledge funding from the Netherlands Organization for Scientific Research (NWO): Biobanking and Biomolecular Research Infrastructure (BBMRI–NL, 184.021.007; 184.033.111); epigenetic data were generated at the Human Genotyping facility (HugeF) of ErasmusMC, the Netherlands. Genotyping was made possible by grants from NWO/SPI 56-464-14192, Genetic Association Information Network (GAIN) of the Foundation for the National Institutes of Health, Rutgers University Cell and DNA Repository (NIMH U24 MH068457-06), the Avera Institute, Sioux Falls (USA) and the National Institutes of Health (NIH R01 HD042157-01A1, MH081802, Grand Opportunity grants 1RC2 MH089951 and 1RC2 MH089995) and European Research Council (ERC-230374). JvD is supported by NWO Large Scale infrastructures, X-Omics (184.034.019). DIB acknowledges the Royal Netherlands Academy of Science Professor Award (PAH/6635).

E-Risk

We would like to thank the E-Risk study members for their participation. The E-Risk Study is funded by the Medical Research Council (G1002190) and the National Institute of Child Health and Human Development (HD077482). Further support was provided by the Jacobs Foundation. The generation of DNA methylation data was supported by a Distinguished Investigator Award to Jonathan Mill by the American Asthma Foundation. This work used a high-performance computing facility partially supported by grant 2016-IDG-1013 (“HARDAC+: Reproducible HPC for Next-generation Genomics”) from the North Carolina Biotechnology Center.

FTC

We would like to thank all the FTC participants, without whom this research would not be possible. This work was supported by the Academy of Finland [213506, 265240, 263278, 312073 to JK, and 297908 to MO], EC FP5 GenomEUtwin (JK), NIH NIH/NHLBI (grant HL104125), EC MC ITN Project EPITRAIN (JK & MO) project and the University of Helsinki Research Funds to MO, Sigrid Juselius Foundation to JK and MO.

TwinsUK

We would like to thank the twins for their participation. TwinsUK was funded by the Wellcome Trust; European Community’s Seventh Framework Programme (FP7/2007-2013) and also receives support from the National Institute for Health Research (NIHR)-funded BioResource, Clinical Research Facility and Biomedical Research Centre based at Guy's and St Thomas' NHS Foundation Trust in partnership with King's College London. The study received additional support from the ESRC (ES/N000404/1 to JTB).

BSGS

We gratefully acknowledge the participation of the twins and their families. This research was supported by NHMRC grants 1010374, 496667 and 1046880, and the National Institutes of Health (NIH) grants GM057091 and GM099568.

NTR-ACTION cohort

We would like to thank the twins and their family members for their participation. The work is supported by the "Aggression in Children: Unraveling gene-environment interplay to inform Treatment and InterventiON strategies" project (ACTION). ACTION received funding from the European Union Seventh Framework Program (FP7/2007-2013) under grant agreement no 602768. The Netherlands Twin Register is supported by grant NWO 480-15-001/674: Netherlands Twin Registry Repository: researching the interplay between genome and environment, the Avera Institute for Human Genetics and by multiple grants from the Netherlands Organization for Scientific Research (NWO), and the Royal Netherlands Academy of Science Professor Award (PAH/6635) to DIB. JvD is supported by the NWO-funded X-omics project (184.034.019).

Supplementary Figures

Figure S1 Comparison of effect sizes in NTR for 243 DMPs across the primary EWAS of MZ versus DZ twins in NTR and sensitivity analyses.

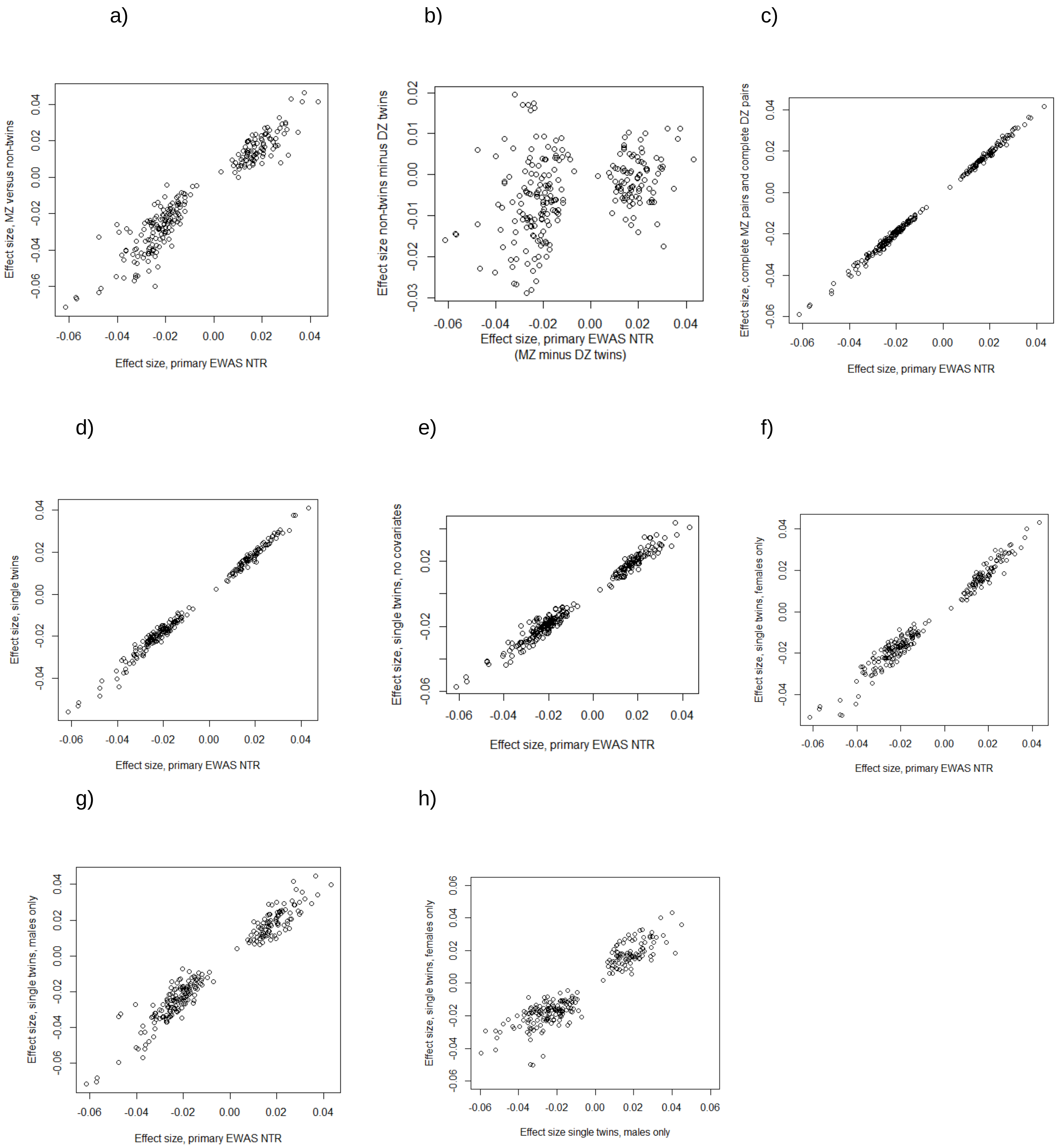

Scatterplots showing the estimates (methylation beta-value difference between MZ twins and controls). X-axis: results from the primary EWAS in NTR (N=1957; controls=DZ twins, participants included: complete DZ pairs and one randomly selected MZ pair). y-axis: Estimate (methylation beta-value difference between MZ twins and controls) from a) Analysis comparing MZ twins to non-twins (parents and siblings), N=1161. b) Analysis comparing non-twins (parents and siblings) to DZ twins, N=1270. c) Analysis in complete MZ pairs and complete DZ pairs (N=2750). c) Analysis in single MZ twins and single DZ twins, randomly selected (N=1538) with a simple linear model (lm function in R). e) Analysis in single MZ twins and single DZ twins, randomly selected (N=1538) with a simple linear model (lm function in R), without any covariates. f) Female-only analysis in single MZ twins and single DZ twins, randomly selected (N=1033) with a simple linear model (lm function in R). g) Male-only analysis in single MZ twins and single DZ twins, randomly selected (N=505) with a simple linear model (lm function in R). h) Effect sizes in males (single twins, N=505, x-axis) versus females (single twins, N=1033, y-axis).

Figure S2 Comparison of effect sizes in NTR versus BSGS for 243 DMPs

a) b)

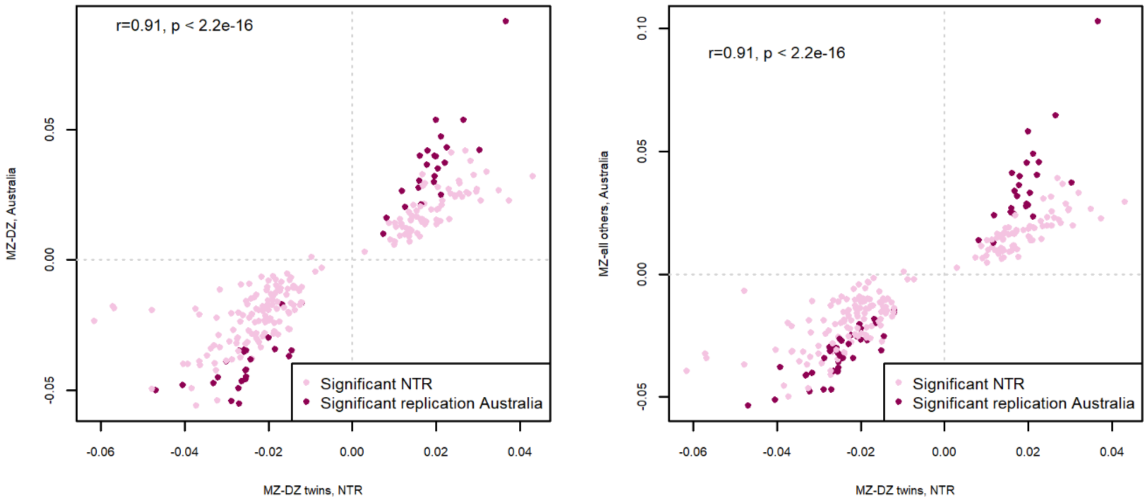

Scatterplot showing the estimates (methylation beta-value difference between MZ twins and controls). X-axis: results from the primary EWAS in NTR (N=1957; controls=DZ twins). y=axis: results from the EWAS in BSGS: a) MZ versus DZ twins (N=356) b) MZ twins versus all other individuals (N=476; all other individuals are DZ twins, siblings, and parents of twins). DMPs that replicate after stringent Bonferroni correction for 243 tests are shown in dark purple.

Figure S3. Twinning-associated DNA methylation and correlation patterns in 11 differentially methylated regions

a)

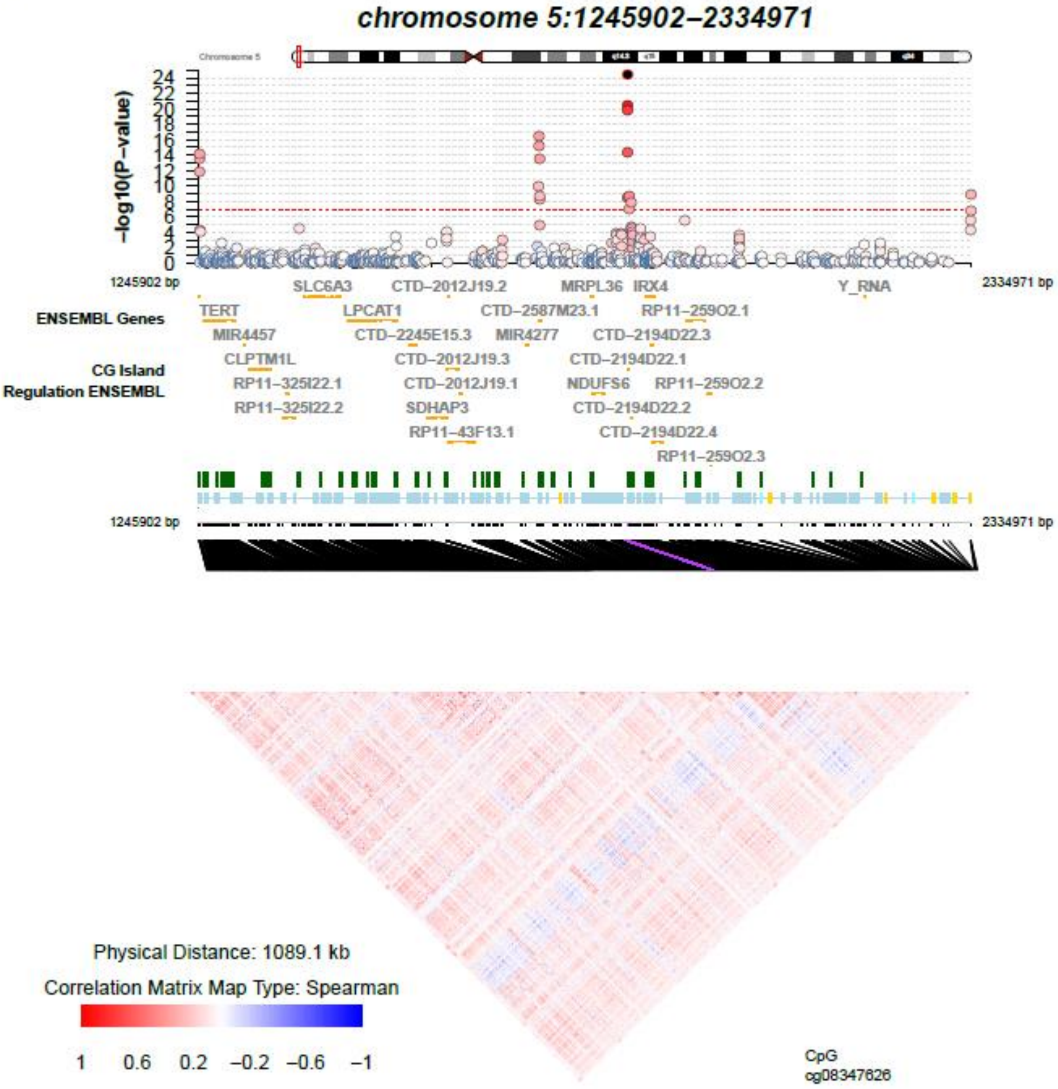

b)

chromosome 5 [140166589-140781179]

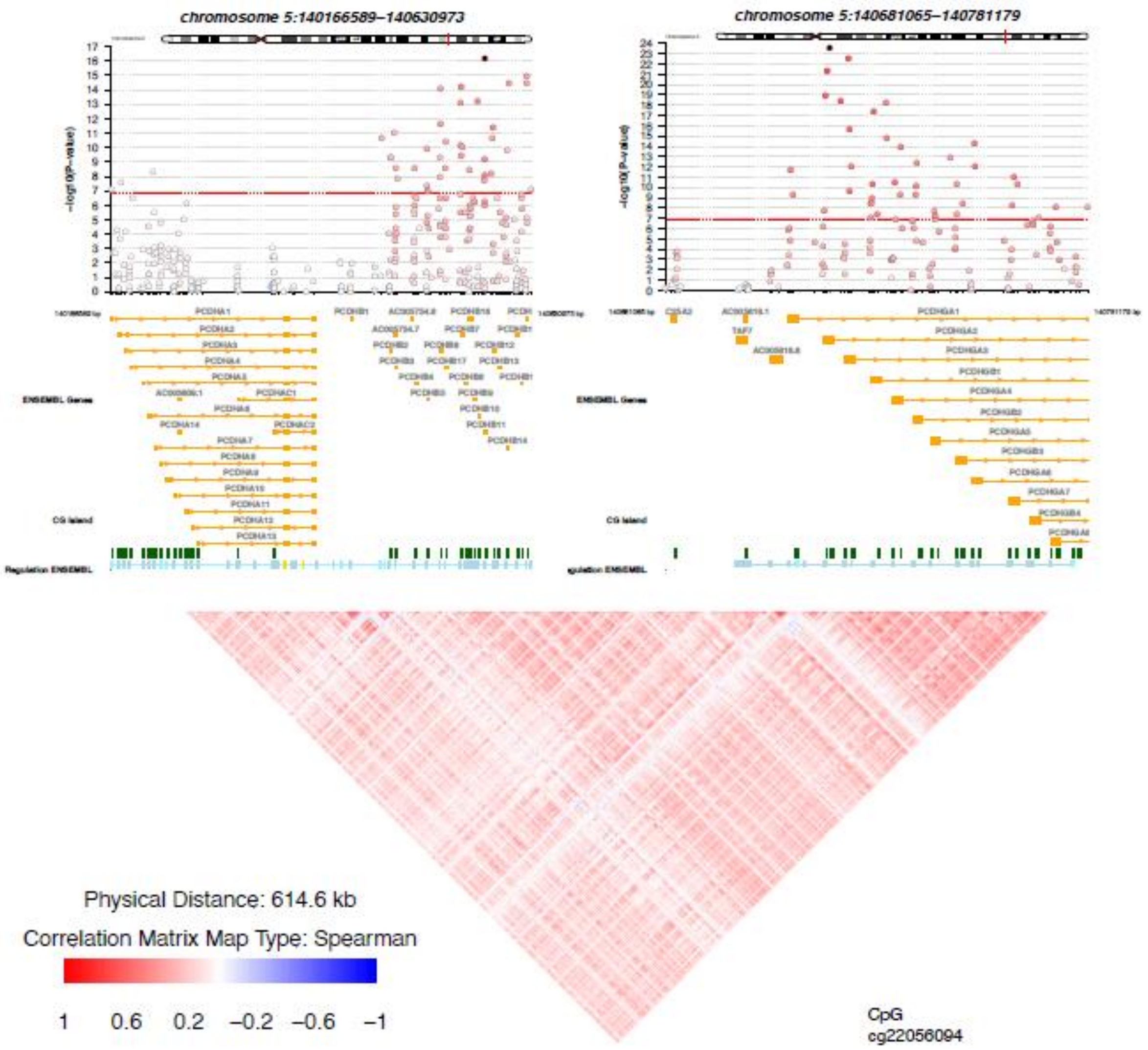

c)

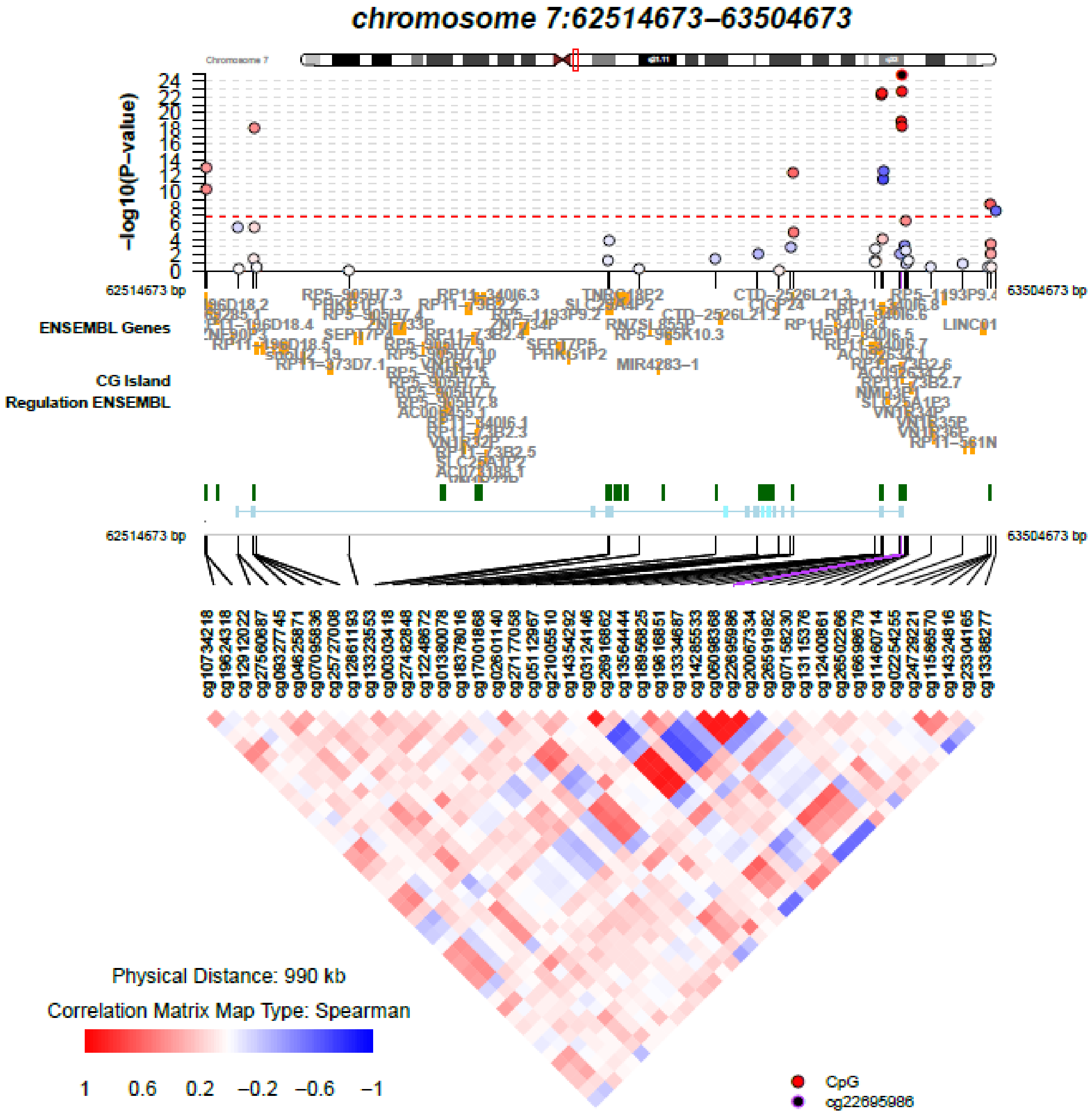
chromosome 7:62514673–63504673
-log10(P-value)
62514673 bp
63504673 bp
ENSEMBL Genes
CG Island
Regulation ENSEMBL
Physical Distance: 990 kb
Correlation Matrix Map Type: Spearman
1 0.6 0.2 −0.2 −0.6 −1
CpG
cg22695986

d)

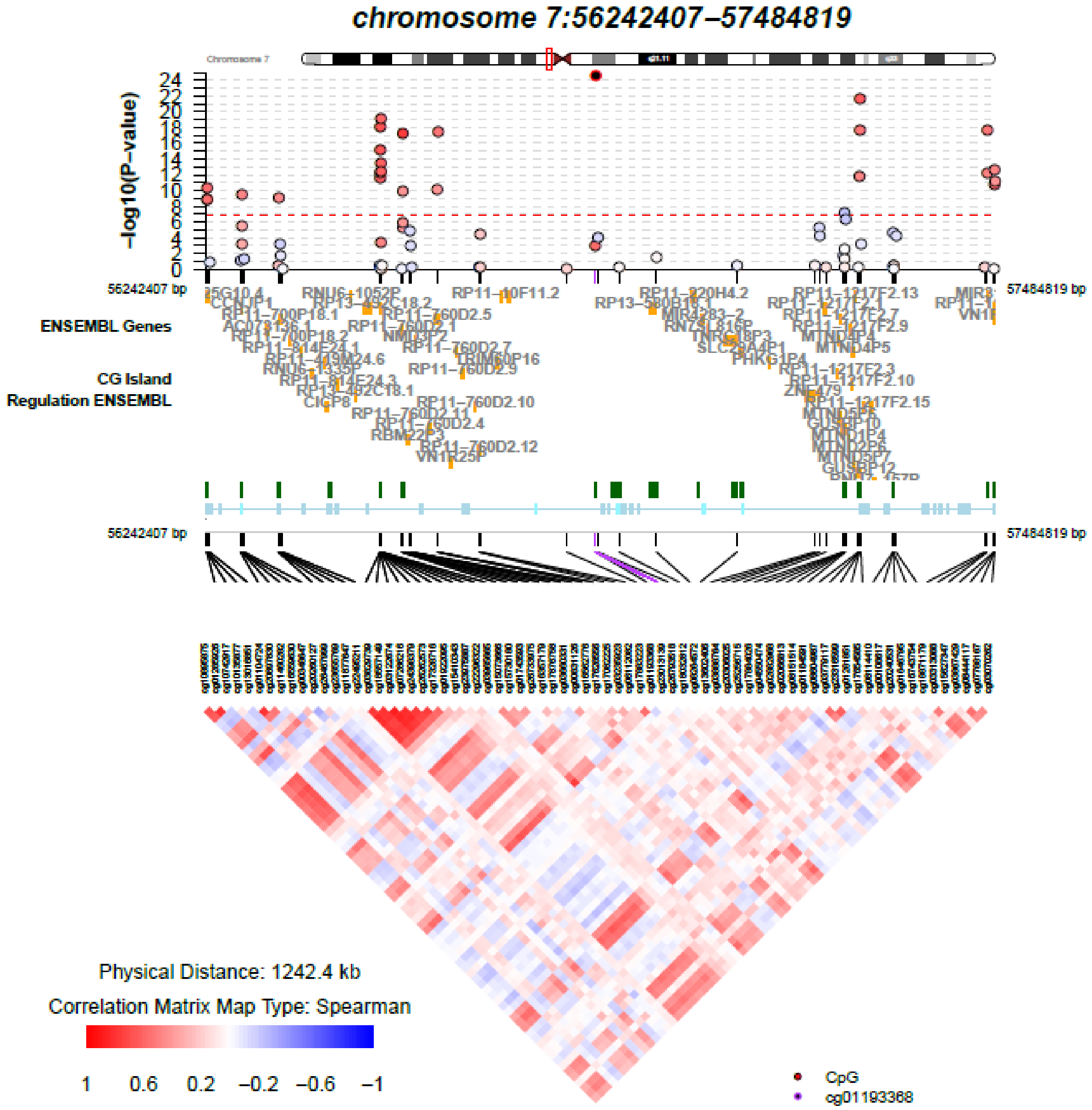
chromosome 7:56242407–57484819
Chromosome 7
−log10(P−value)
56242407 bp
57484819 bp
ENSEMBL Genes
CG Island
Regulation ENSEMBL
RP11−10F11.2
RP11−820H4.2
RP11−1217F2.13
RP11−760D2.5
RP11−760D2.7
RP11−760D2.10
RP11−760D2.12
RP11−1217F2.9
RP11−1217F2.10
RP11−1217F2.15
Physical Distance: 1242.4 kb
Correlation Matrix Map Type: Spearman
1
0.6
0.2
−0.2
−0.6
−1
CpG
cg01193368

e)

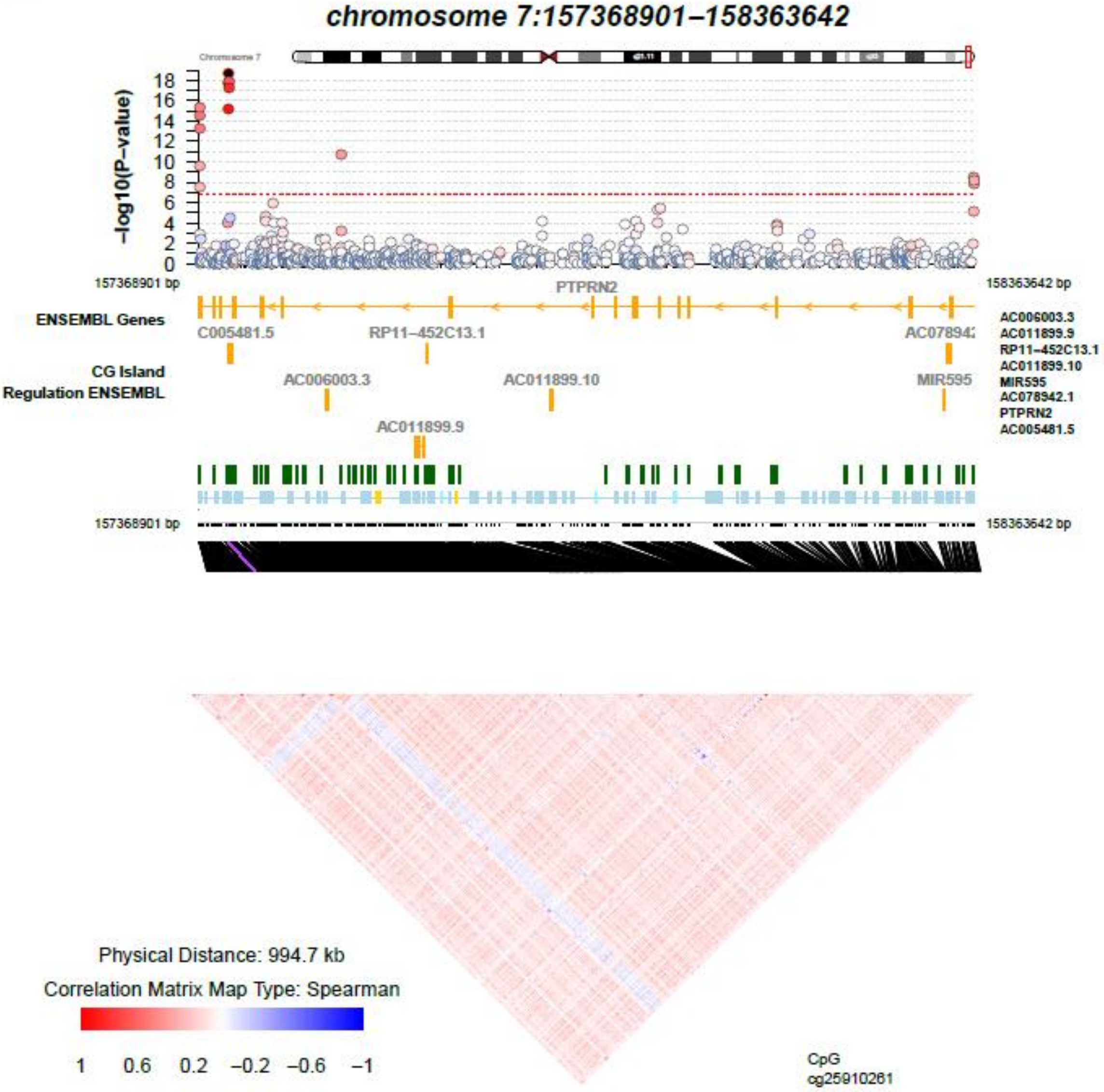
chromosome 7:157368901–158363642
Chromosome 7
−log10(P−value)
18
16
14
12
10
8
6
4
2
0
157368901 bp
158363642 bp
PTPRN2
ENSEMBL Genes
C005481.5
RP11−452C13.1
AC07894;
CG Island
Regulation ENSEMBL
AC006003.3
AC011899.10
MIR595
AC011899.9
AC006003.3
AC011899.9
RP11−452C13.1
AC011899.10
MIR595
AC078942.1
PTPRN2
AC005481.5
Physical Distance: 994.7 kb
Correlation Matrix Map Type: Spearman
1
0.6
0.2
−0.2
−0.6
−1
CpG
cg25910261

f)

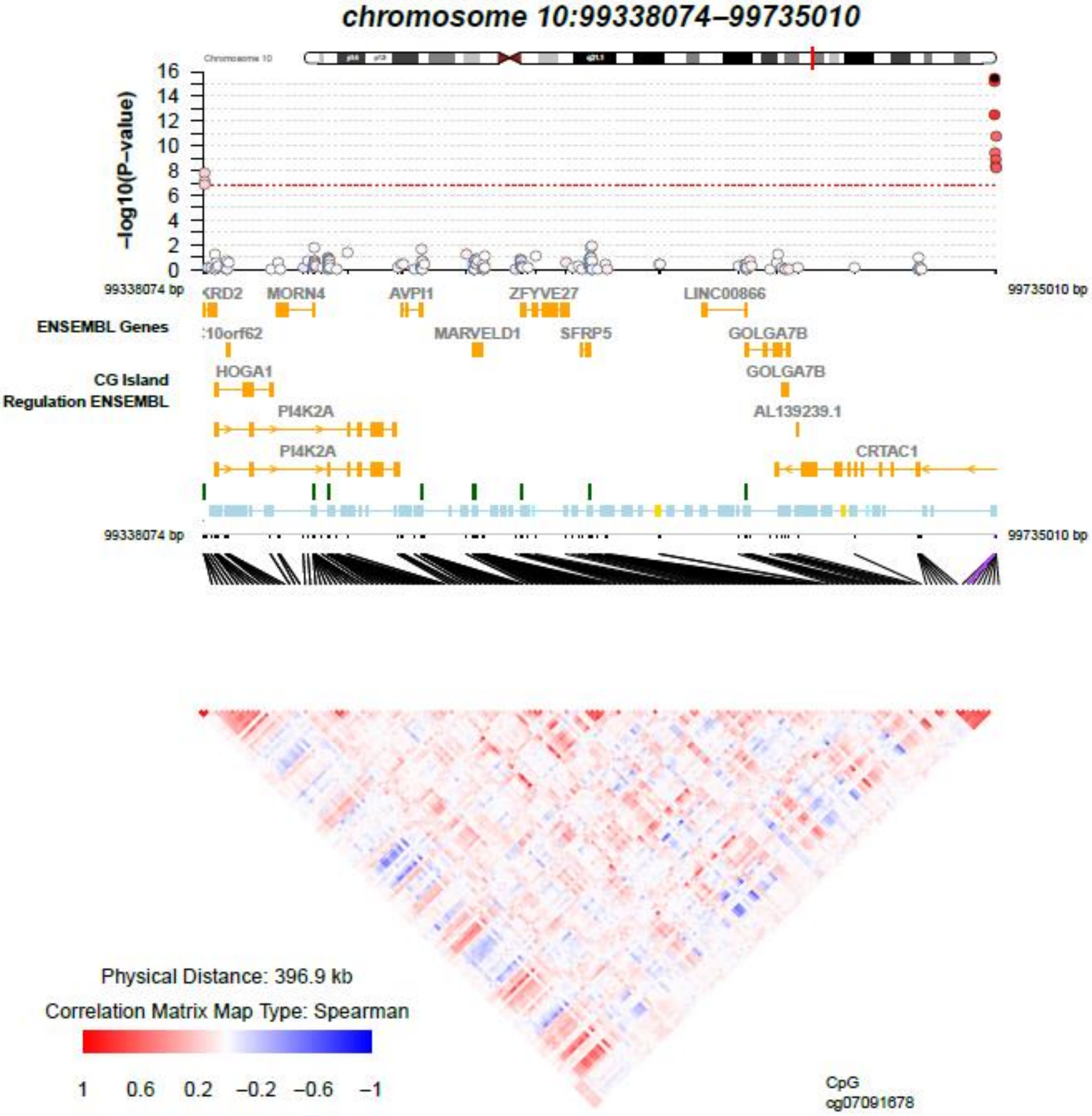

chromosome 10:99338074–99735010
Chromosome 10
−log10(P−value)
16
14
12
10
8
6
4
2
0
99338074 bp
99735010 bp
ENSEMBL Genes
CG Island
Regulation ENSEMBL
KRD2
MORN4
AVPI1
ZFYVE27
LINC00866
:10orf62
MARVELD1
SFRP5
GOLGA7B
HOGA1
GOLGA7B
PI4K2A
AL139239.1
PI4K2A
CRTAC1
Physical Distance: 396.9 kb
Correlation Matrix Map Type: Spearman
1 0.6 0.2 −0.2 −0.6 −1
CpG
cg07091678

g)

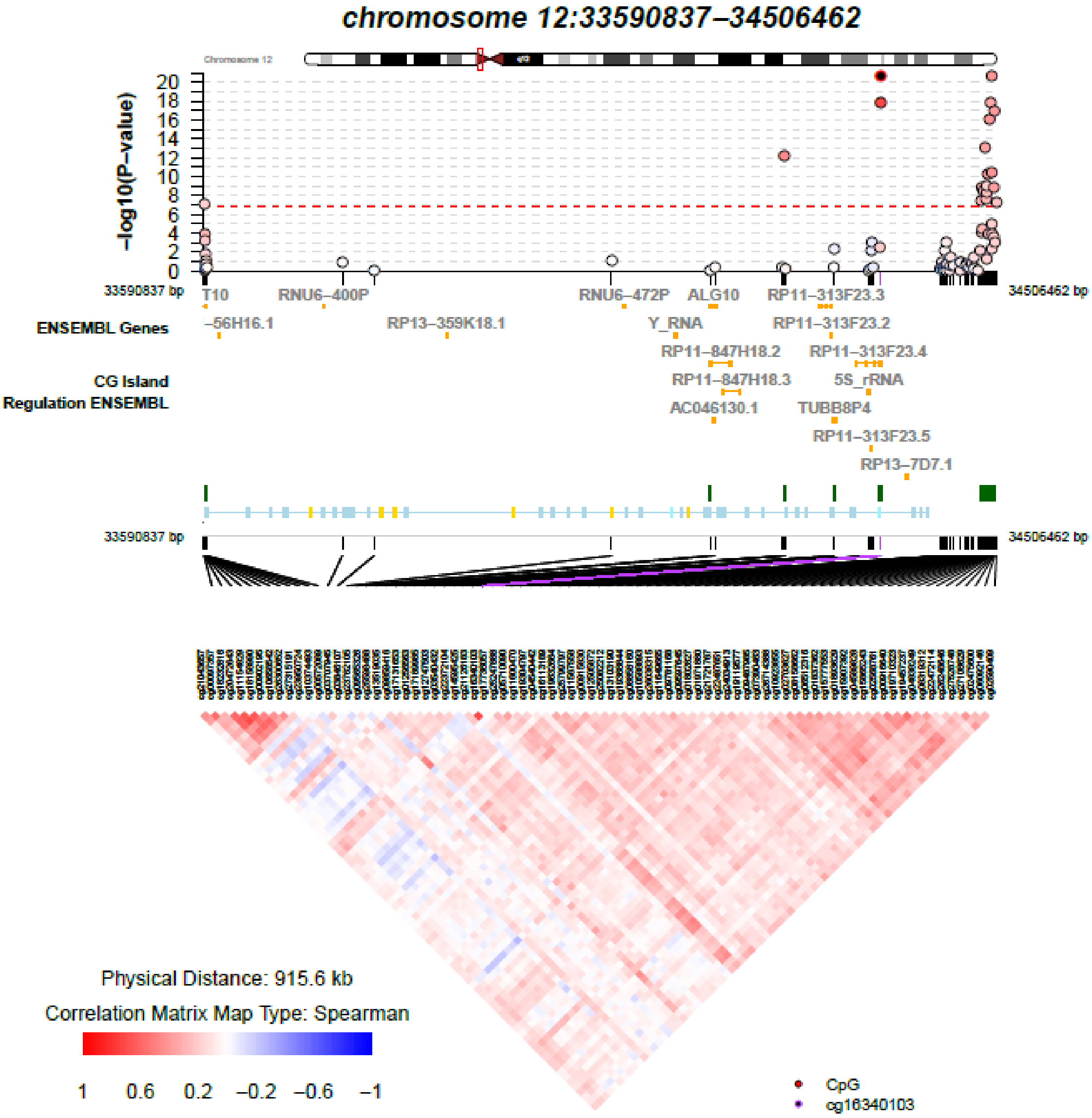
chromosome 12:33590837–34506462
Chromosome 12
−log10(P−value)
20
18
16
14
12
10
8
6
4
2
0
33590837 bp
34506462 bp
ENSEMBL Genes
T10
RNU6–400P
RNU6–472P
ALG10
RP11–313F23.3
–56H16.1
RP13–359K18.1
Y_RNA
RP11–313F23.2
RP11–847H18.2
RP11–313F23.4
RP11–847H18.3
5S_rRNA
AC046130.1
TUBB8P4
RP11–313F23.5
RP13–7D7.1
CG Island
Regulation ENSEMBL
Physical Distance: 915.6 kb
Correlation Matrix Map Type: Spearman
1
0.6
0.2
−0.2
−0.6
−1
CpG
cg16340103

h)

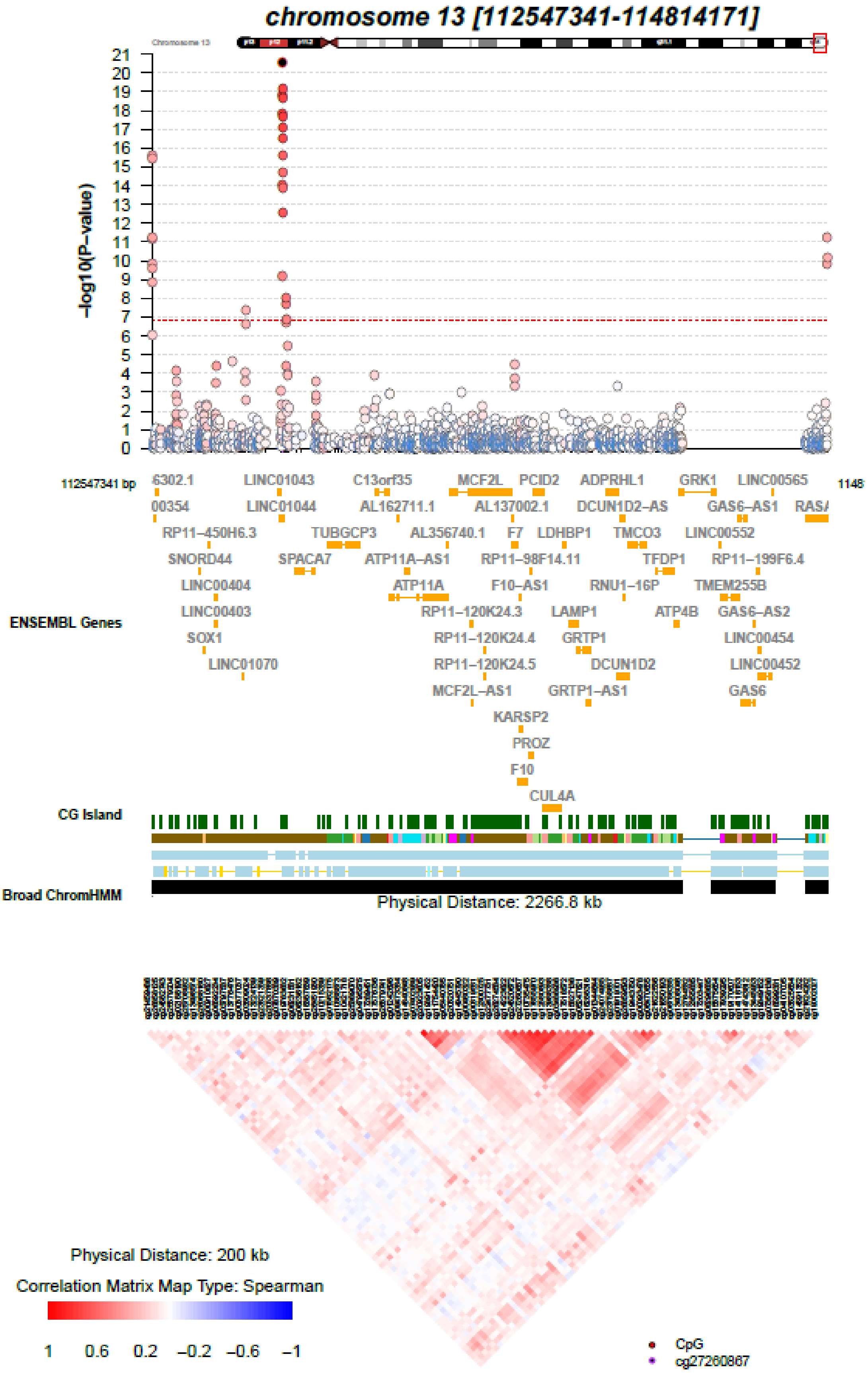

chromosome 13 [112547341-114814171]
Chromosome 13
-log10(P-value)
0
1
2
3
4
5
6
7
8
9
10
11
12
13
14
15
16
17
18
19
20
21
112547341 bp
1148
ENSEMBL Genes
6302.1
00354
RP11-450H6.3
SNORD44
LINC00404
LINC00403
SOX1
LINC01070
LINC01043
LINC01044
TUBGCP3
SPACA7
C13orf35
AL162711.1
AL356740.1
ATP11A-AS1
ATP11A
MCF2L
AL137002.1
F7
RP11-98F14.11
F10-AS1
RP11-120K24.3
RP11-120K24.4
RP11-120K24.5
MCF2L-AS1
KARSP2
PROZ
F10
CUL4A
PCID2
LDHBP1
LAMP1
GRTP1
GRTP1-AS1
ADPRHL1
DCUN1D2-AS
TMCO3
TFDP1
RNU1-16P
DCUN1D2
ATP4B
GRK1
LINC00552
GAS6-AS1
RP11-199F6.4
TMEM255B
GAS6-AS2
LINC00454
LINC00452
GAS6
LINC00565
RAS/
CG Island
Broad ChromHMM
Physical Distance: 2266.8 kb
Physical Distance: 200 kb
Correlation Matrix Map Type: Spearman
1
0.6
0.2
-0.2
-0.6
-1
CpG
cg27260867

i)

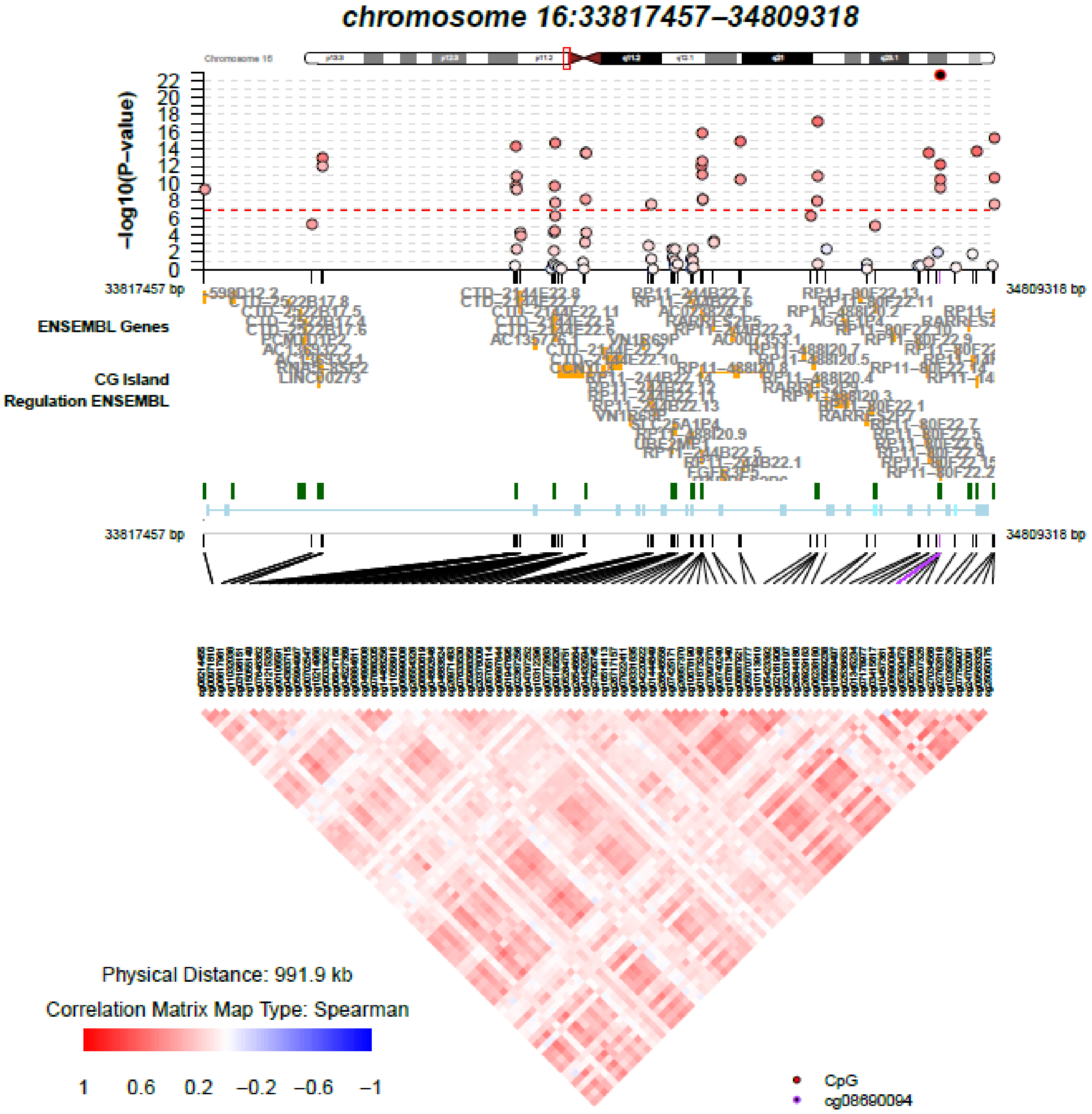

chromosome 16:33817457–34809318
−log10(P−value)
33817457 bp
34809318 bp
ENSEMBL Genes
CG Island
Regulation ENSEMBL
Physical Distance: 991.9 kb
Correlation Matrix Map Type: Spearman
1 0.6 0.2 −0.2 −0.6 −1
CpG
cg08690094

j)

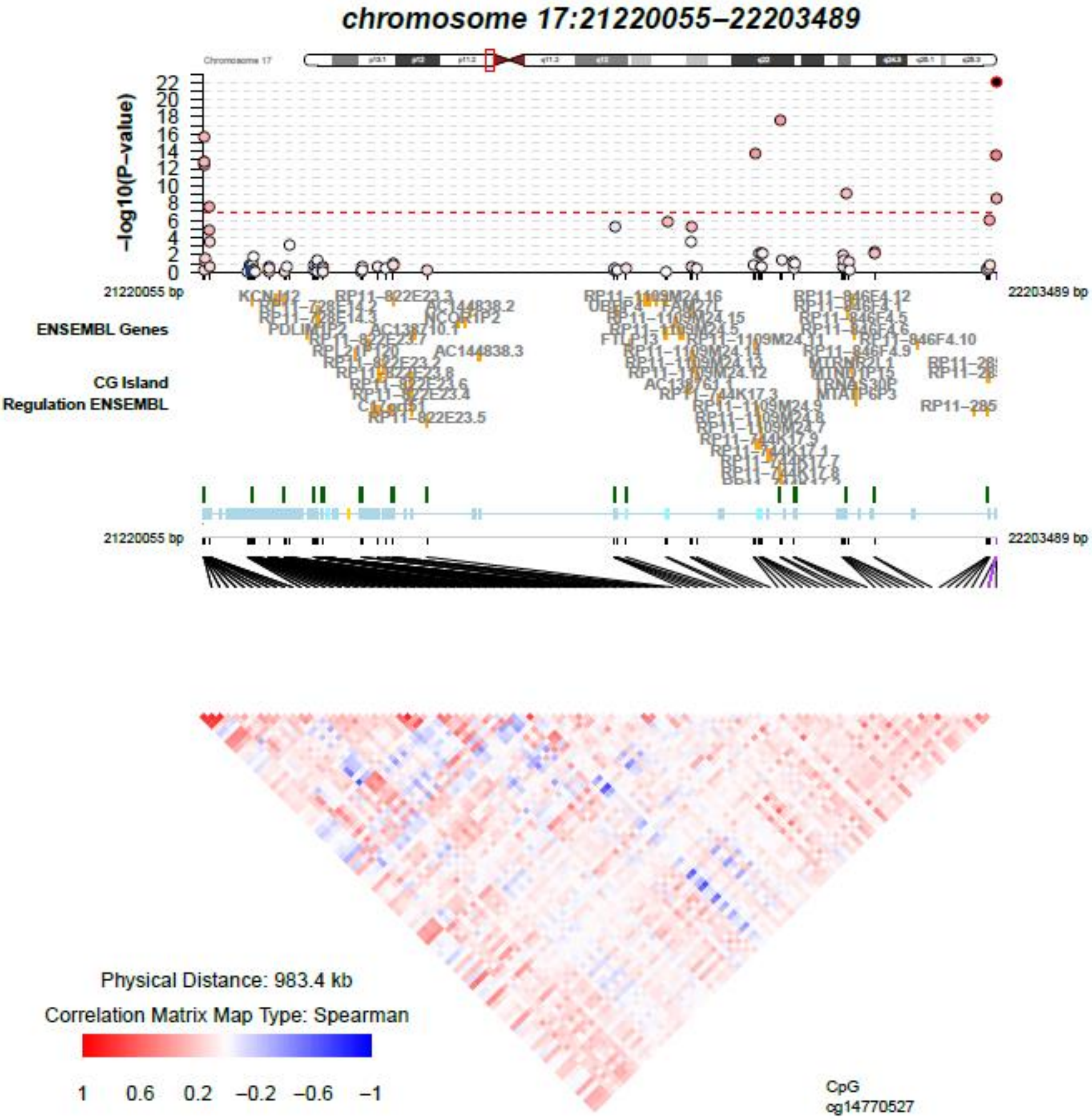

chromosome 17:21220055–22203489
-log10(P-value)
21220055 bp
22203489 bp
ENSEMBL Genes
CG Island
Regulation ENSEMBL
Physical Distance: 983.4 kb
Correlation Matrix Map Type: Spearman
1 0.6 0.2 −0.2 −0.6 −1
CpG
cg14770527

k)

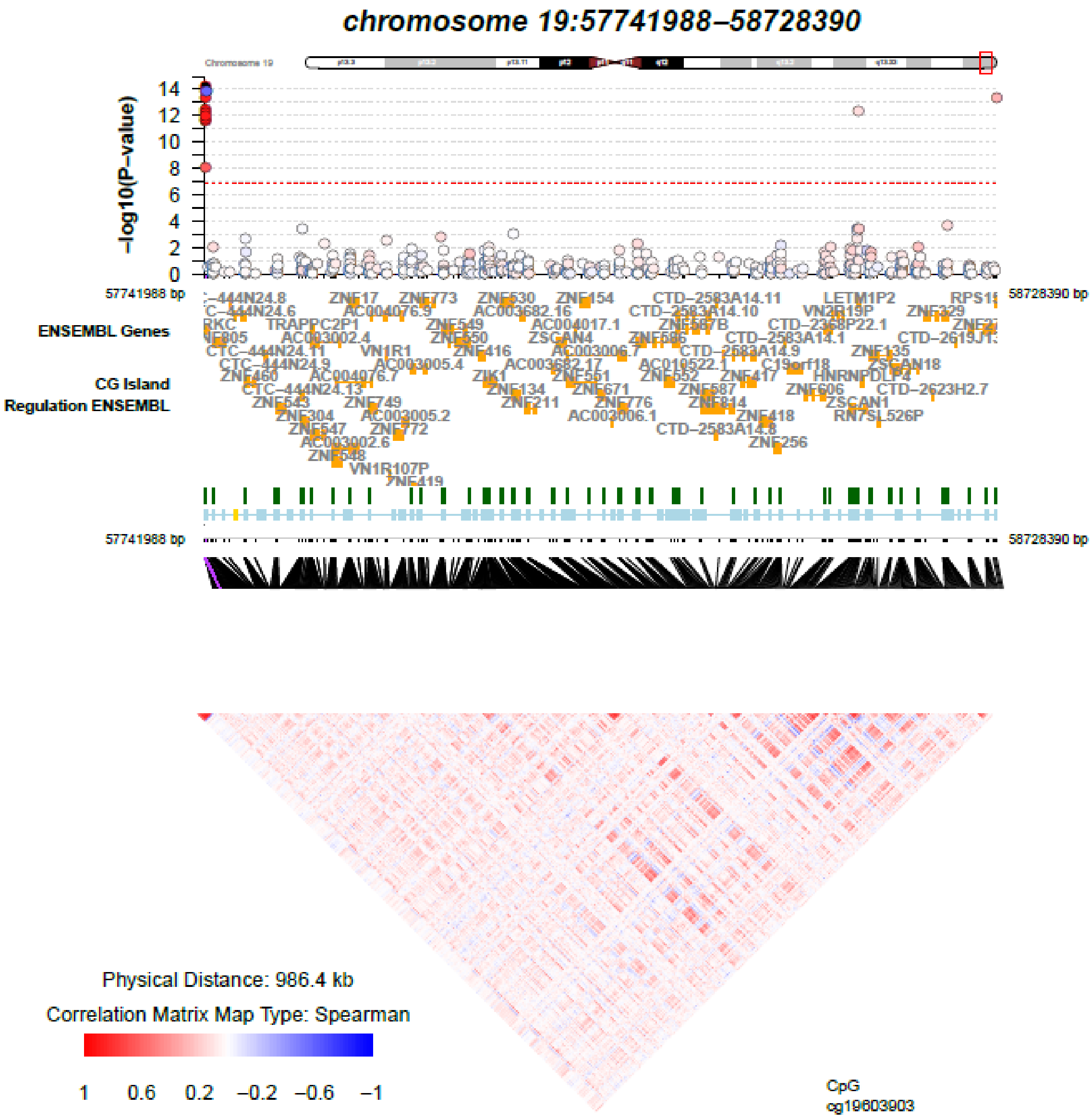
chromosome 19:57741988–58728390
Chromosome 19
−log10(P−value)
14
12
10
8
6
4
2
0
57741988 bp
58728390 bp
ENSEMBL Genes
CG Island
Regulation ENSEMBL
ZNF17
ZNF773
ZNF530
ZNF154
CTD−2583A14.11
LETM1P2
TRAPPC2P1
ZNF549
AC003682.16
AC004076.9
CTD−2583A14.10
VN2R19P
ZNF329
ZNF550
AC004017.1
ZNF587B
CTD−2368P22.1
AC003002.4
ZSCAN4
ZNF586
CTD−2583A14.1
VN1R1
ZNF416
AC003006.7
CTD−2583A14.9
ZNF135
CTC−444N24.11
CTC−444N24.9
AC003005.4
AC003682.17
AC010522.1
C19orf18
ZSCAN18
ZNF460
AC004076.7
ZIK1
ZNF551
ZNF552
ZNF417
HNRNPDLP4
CTC−444N24.13
ZNF134
ZNF671
ZNF587
ZNF606
CTD−2623H2.7
ZNF543
ZNF749
ZNF211
ZNF776
ZNF814
ZSCAN1
ZNF304
AC003005.2
AC003006.1
ZNF418
RN7SL526P
ZNF547
ZNF772
CTD−2583A14.8
AC003002.6
ZNF256
ZNF548
VN1R107P
ZNF419
Physical Distance: 986.4 kb
Correlation Matrix Map Type: Spearman
1
0.6
0.2
−0.2
−0.6
−1
CpG
cg19603903

a-k) Plots of exemplary regions that contain multiple CpGs significantly associated with MZ twinning. Regions were defined by selecting a window of 1 Mb around each differentially DMP with the lowest p-value. Plots were created for all regions with 10 or more significant CpGs in this window. The top panel of each plot shows the EWAS meta-analysis p-values for all CpGs in the window, with the most strongly associated CpG highlighted. CpGs above the red horizontal line are epigenome-wide significant in the meta-analysis (total sample size = 5,723). The middle panel shows the genomic coordinates (genome build GRCh37/hg19) and the functional annotation of the region: the ENSEMBL Genes track shows the genes in the genomic region (orange); the CpG Island track shows the location of CpG islands (green); the Regulation ENSEMBL track shows regulatory regions. The bottom panel shows the Spearman correlation between methylation levels of CpGs in the window based on whole blood Illumina 450k methylation data from the NTR (N=3,089 samples). a) chromosome 5:1245902-2334971, 782 CpGs, 18 significant CpGs, mean correlation between significant CpGs 0.328 [range=0.053; 0.919]. b) chromosome 5:140166589-140781179, 476 CpGs, 79 significant CpGs, mean correlation between significant CpGs: 0.283 [range=-0.029; 0.719]. c) chromosome 7:62514673-63504673, 44 CpGs, 14 significant CpGs, mean correlation between significant CpGs 0.195 [range=-0.690; 0.893]. d) chromosome 7:56242407–57484819, 73 CpGs, 26 significant CpGs, mean correlation between significant CpGs 0.446 [range=-0.228; 0.866]. e) chromosome 7:157368901-158363642, 844 CpGs, 14 significant CpGs, mean correlation between significant CpGs 0.420 [range=0.144; 0.954]. f) chromosome 10:99338074-99735010, 153 CpGs, 10 significant CpGs, mean correlation between significant CpGs 0.474 [range=0.106; 0.945]. g) chromosome 12:33590837-34506462, 82 CpGs, 19 significant CpGs, mean correlation between significant CpGs 0.317 [range=0.068;0.700]. h) chromosome 13:112547341-114814171, 1688 CpGs, 27 significant CpGs, mean correlation between significant CpGs 0.400 [range=0.035; 0.931]. i) chromosome 16: 33817457-34809318, 1688 CpGs, 34 significant CpGs, mean correlation between significant CpGs 0.331 [range=0.138; 0.563]. j) chromosome 17:21220055-22203489, 98 CpGs, 11 significant CpGs, mean correlation between significant CpGs 0.381 [range=0.207;0.850]. k) chromosome 19:57741988-58728390, 480 CpGs, 12 significant CpGs, mean correlation between significant CpGs 0.444 [range=-0.592; 0.978].

Figure S4 Average methylation level in blood and longitudinal correlation for 834 MZ-DMPs.

a)

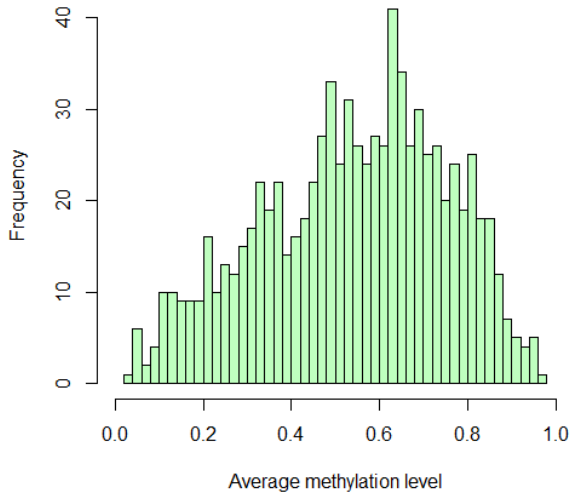

b)

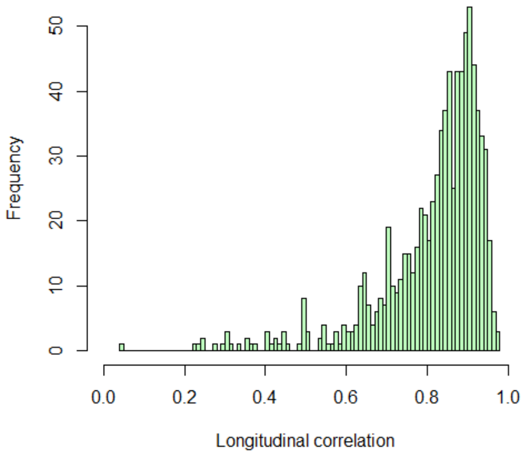

a)Histogram of the average DNA methylation level in blood at 834 MZ-DMPs, based on data from the Netherlands Twin Register (N= 3057 individuals). b) Histogram of the correlations between longitudinal peripheral blood DNA methylation levels collected with an interval of on average 5 years, based on data from the Netherlands Twin Register (N= 31 individuals).

Figure S5 Heritability and SNP heritability for 834 MZ-DMPs based on whole blood methylation data.

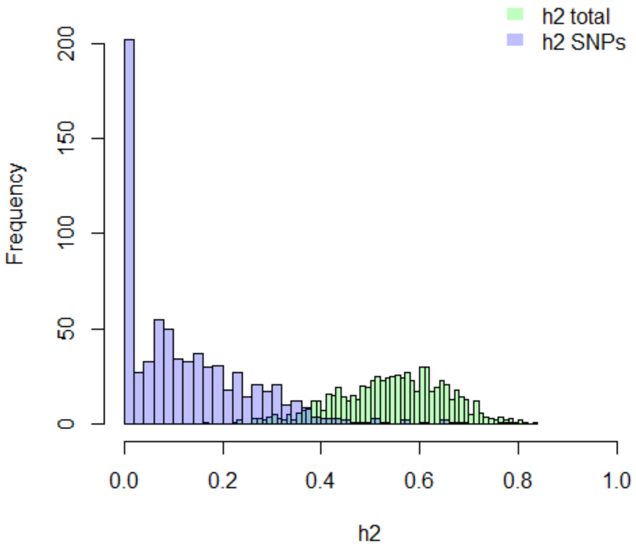

Histogram of the total heritability (h^2) and SNP heritability (h^2 SNPs) of DNA methylation level in blood at 834 MZ-DMPs, based on data from the Netherlands Twin Register (N= 2,603 individuals).

Figure S6 Twin correlations and ADE twin model estimates for 834 MZ-DMPs based on whole blood methylation data.

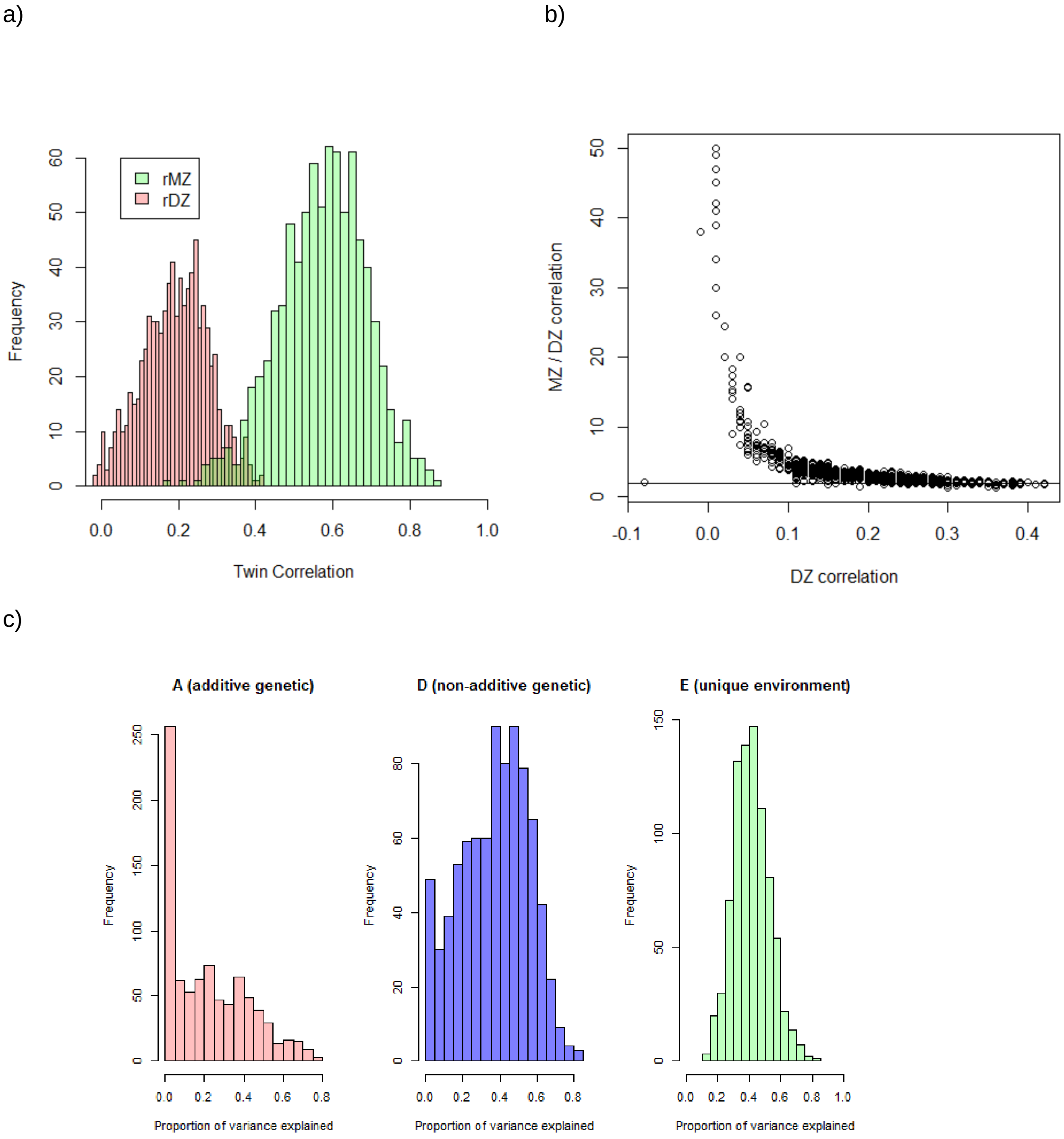

Twin correlations and ADE model estimates for DNA methylation level in blood at 834 MZ-DMPs, based on data from the Netherlands Twin Register. a) Histograms of the correlation between DNA methylation levels of monozygotic twins (rMZ, N MZ pairs= 769), and dizygotic twins (rDZ, N DZ pairs=424). b) Scatterplot of the DZ twin correlation (x-axis) versus the ratio of the twin correlations (rMZ/rDZ; y-axis). c) Proportion of variance in DNA methylation level explained by additive genetic effects, non-additive genetic effects, and unique environment.

Figure S7 Within-pair differences in MZ pairs at ten exemplary MZ-DMPs based on whole blood methylation data.

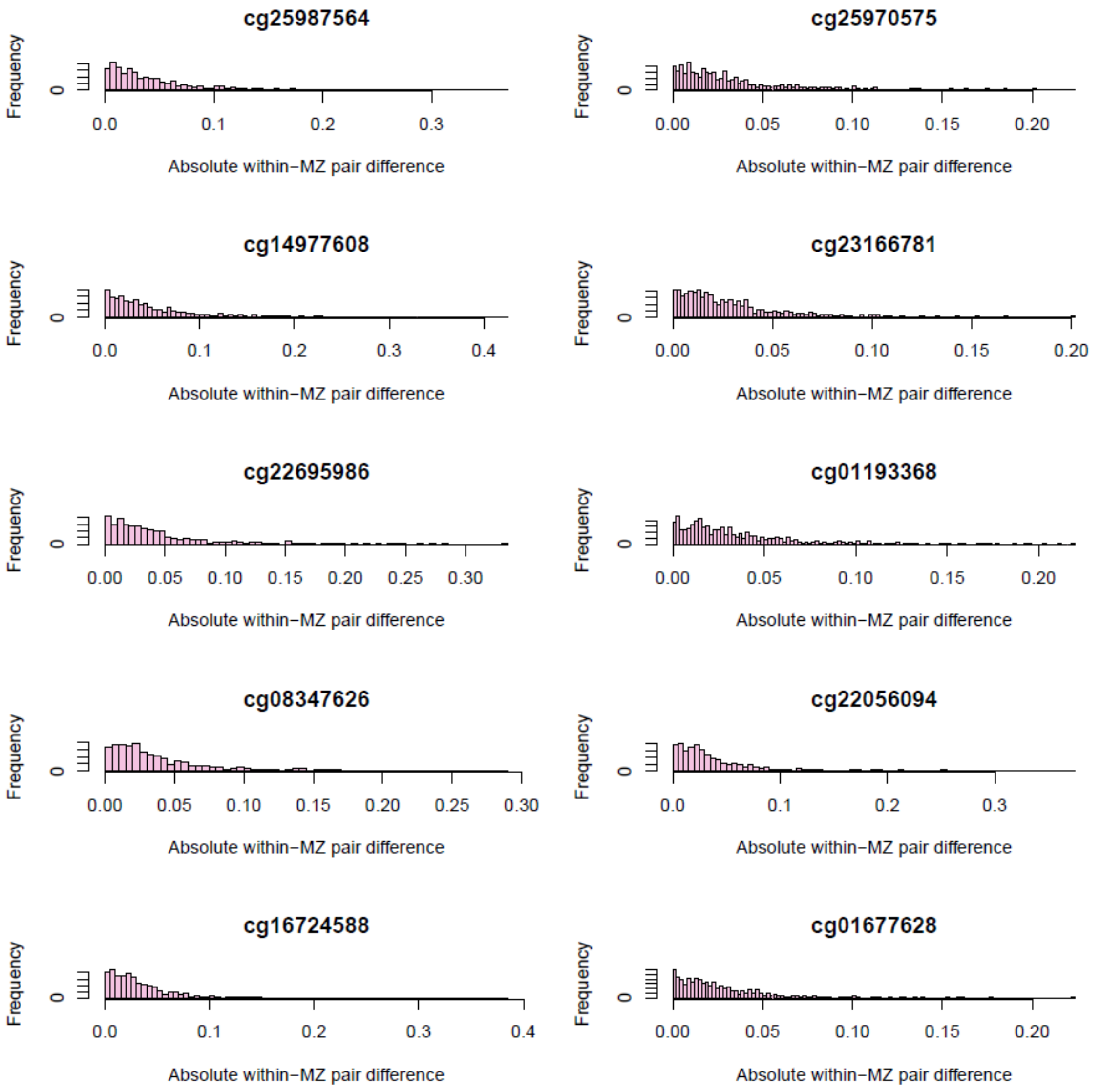

Histograms of absolute within MZ pair differences in DNA methylation levels are shown for ten exemplary CpGs that were randomly selected from the total set of 834 MZ-DMPs, and illustrate the skewed distribution of with-pair differences. Within-pair differences were calculated for 761 MZ twin pairs, based on whole blood methylation data from the Netherlands Twin Register. The figures show absolute within-pair differences of residual methylation levels, which were obtained after adjusting methylation beta-values for covariates.

Figure S8 MZ twin scatterplots (twin 1 versus twin 2) of ten exemplary MZ-DMPs based on whole blood methylation data.

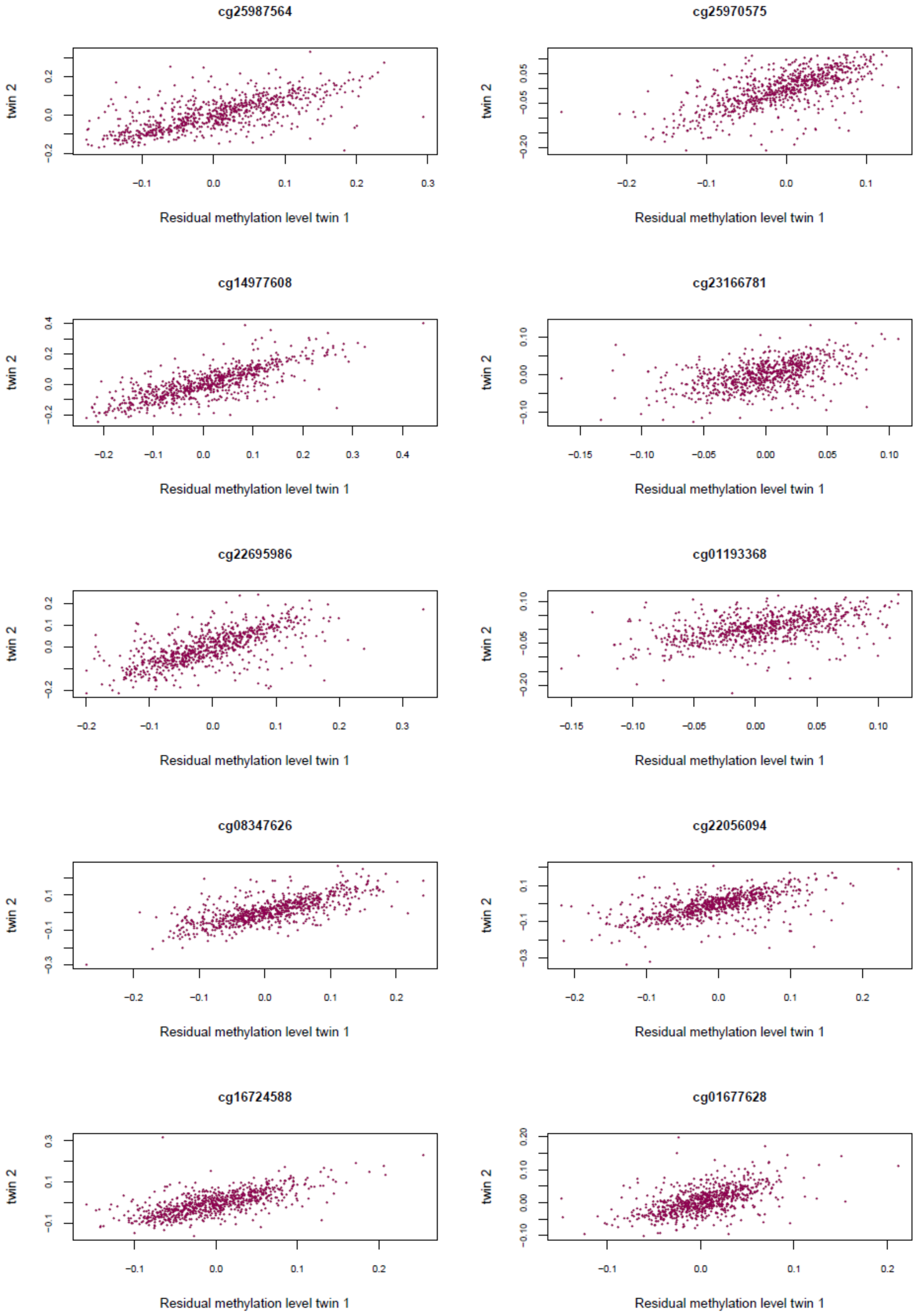

Scatterplots are shown for ten exemplary CpGs that were randomly selected from the total set of 834 MZ-DMPs. Data are shown for 761 MZ twin pairs (each dot represents one twin pair), based on whole blood methylation data from the Netherlands Twin Register. The figures show residual methylation levels, which were obtained after adjusting methylation beta-values for covariates. The value of twin 1 is shown on the x-axis and the value of twin 2 is shown on the y-axis.

Figure S9 Within-pair and between-pair differences at 834 MZ-DMPs

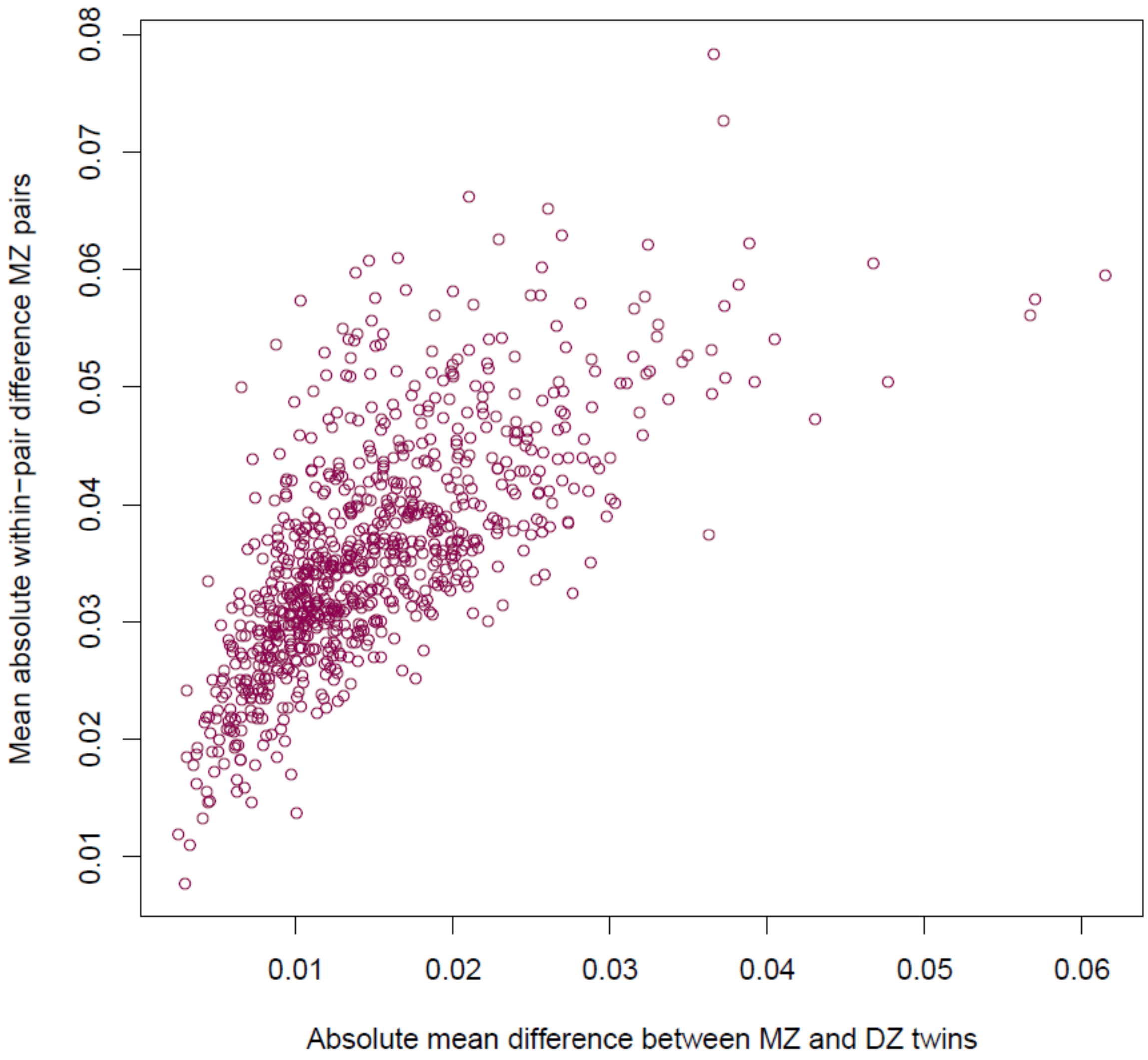

The x-axis shows the absolute mean difference in DNA methylation level between MZ and DZ twins and the y-axis shows the absolute within-pair dfiference in MZ twins at 834 MZ-DMPs, based on whole blood methylation data from the Netherlands Twin Register. Mean differences between MZ and DZ twins were taken from the primary EWAS analysis in NTR. Mean differences within MZ pairs were calculated on residual methylation levels, which were obtained after adjusting methylation beta-values for covariates.

Figure S10 Distribution of within-pair differences in MZ pairs at 834 MZ-DMPs

a)

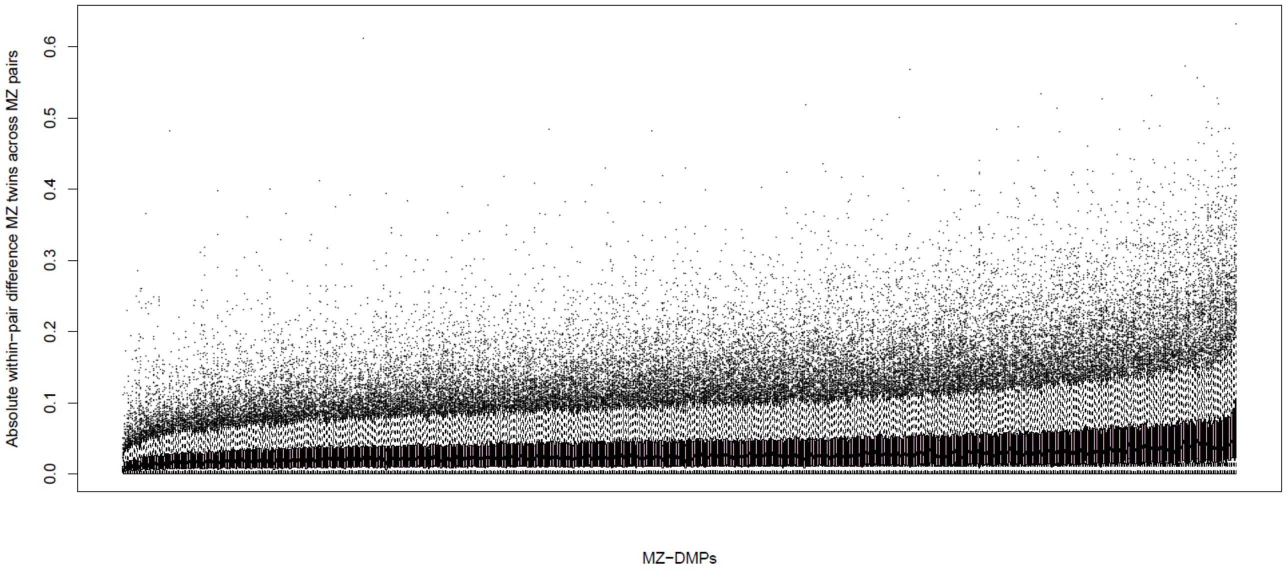

b)

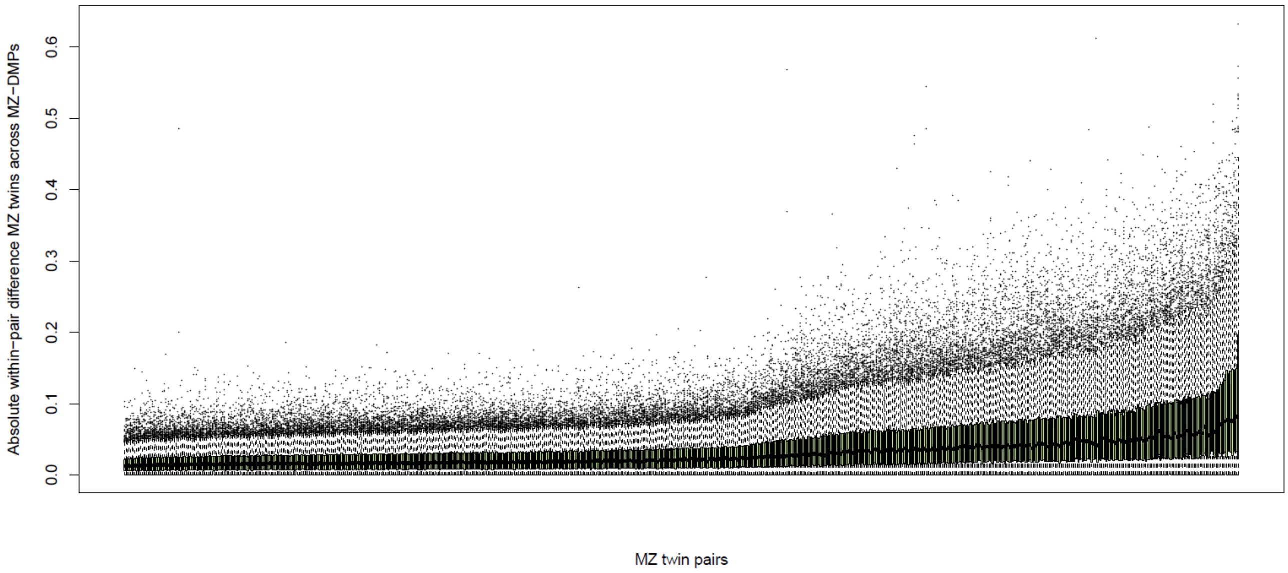

Figure a shows boxplots of within-pair differences across pairs for each CpG. Figure b shows boxplots of within-pair differences across CpGs for each MZ pair. Within-pair differences were calculated for 761 MZ twin pairs, based on whole blood methylation data from the Netherlands Twin Register. The figures show absolute within-pair differences of residual methylation levels, which were obtained after adjusting methylation beta-values for covariates. Thick horizontal lines within boxes denote the median, box edges show the 25th and 75th percentiles, whiskers denote 1.5×interquartile range (IQR), and dots any datapoints outside this range.

Figure S11 Twin correlations in buccal cell DNA methylation data from an independent group of children from the Netherlands Twin Register.

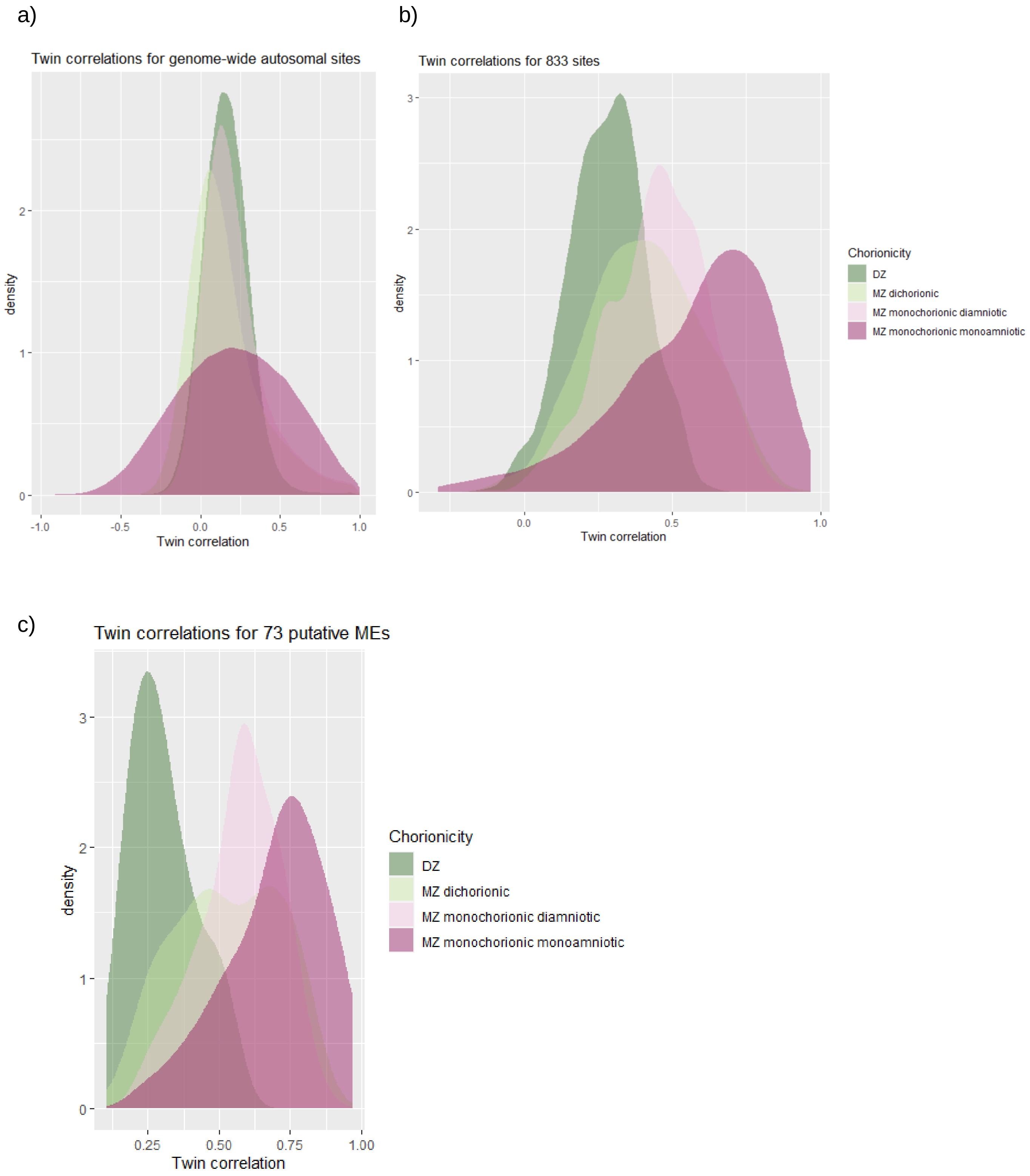

a)Density plots of twin correlations for genome-wide autosomal methylation sites. b) Twin correlations for the 833 MZ-DMPs that were present in the buccal DNA methylation dataset. c) Twin correlations for previously published putative metastable epi-alleles. MZ= Monozygotic twins. DZ=Dizygotic twins. ME=metastable epi-alleles.

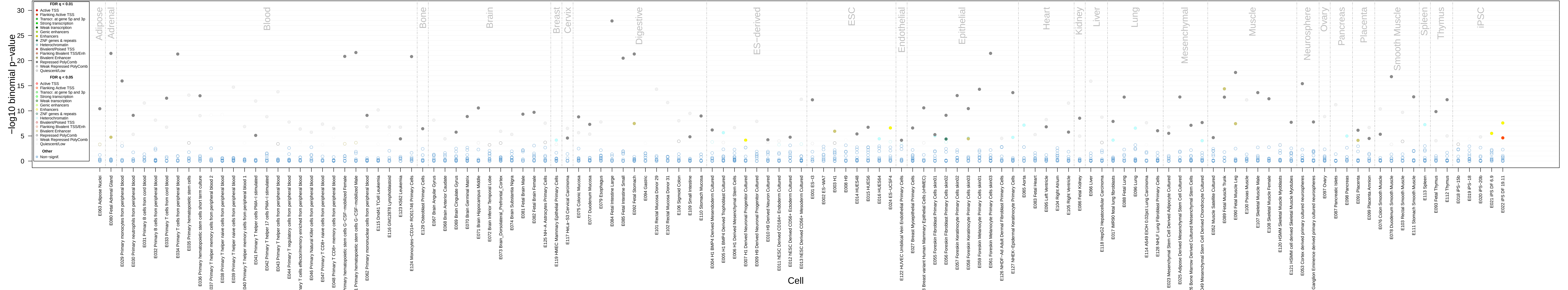
−log10 binomial p−value
Cell
Adipose
Adrenal
Blood
Bone
Brain
Breast
Cervix
Digestive
ES−deriv
ESC
Endothelial
Epithelial
Heart
Kidney
Liver
Lung
Mesenchymal
Muscle
Neurosphere
Ovary
Pancreas
Placenta
Smooth Muscle
Spleen
Thymus
iPSC
FDR q < 0.01
FDR q < 0.05
Other
Non−signif.

Figure S12 Chromatin state enrichment analysis of MZ-hypomethylated sites (previous page**)**

Results from the enrichment analysis of 15 Epigenomic Roadmap Chromatin States for MZ-hypomethylated sites.

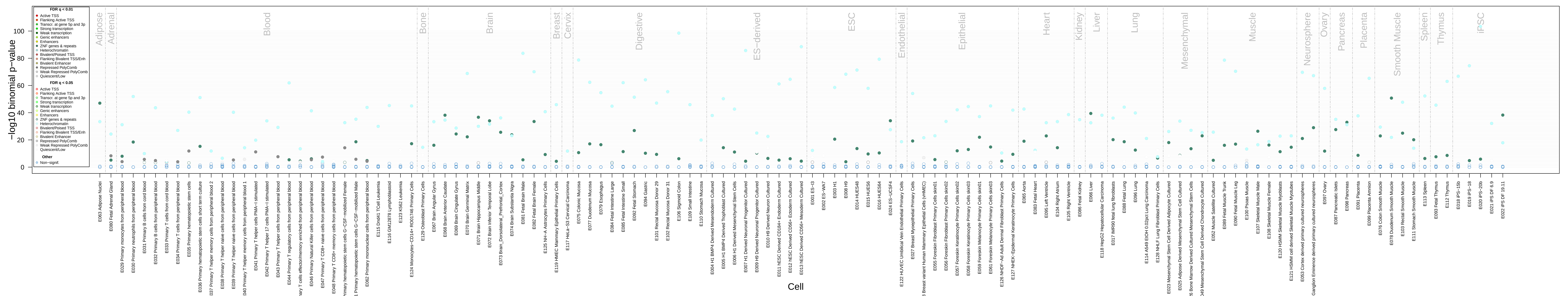
−log10 binomial p−value
Cell
Adipose
Adrenal
Blood
Bone
Brain
Breast
Cervix
Digestive
ES−deriv
ESC
Endothelial
Epithelial
Heart
Kidney
Liver
Lung
Mesenchymal
Muscle
Neurosphere
Ovary
Pancreas
Placenta
Smooth Muscle
Spleen
Thymus
iPSC
FDR q < 0.01
Active TSS
Flanking Active TSS
Transcr. at gene 5p and 3p
Strong transcription
Weak transcription
Genic enhancers
Enhancers
ZNF genes & repeats
Heterochromatin
Bivalent/Poised TSS
Flanking Bivalent TSS/Enh
Bivalent Enhancer
Repressed PolyComb
Weak Repressed PolyComb
Quiescent/Low
FDR q < 0.05
Other
Non−signif.

Figure S13 Chromatin state enrichment analysis of MZ-hypermethylated sites (previous page**)**

Results from the enrichment analysis of 15 Epigenomic Roadmap Chromatin States for MZ-hypermethylated sites.

Figure S14 QQ-plots from the EWAS meta-analysis of MZ versus DZ twins, highlighting methylation sites within imprinted DMRs.

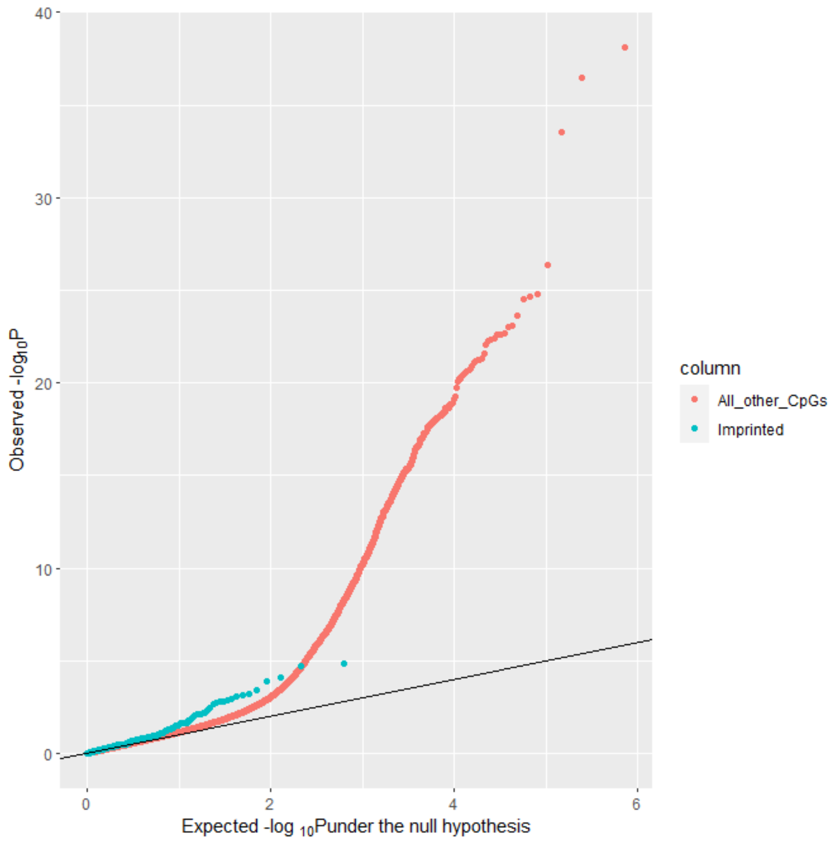

P-values from the EWAS meta-analysis (sample size = 5,723) are shown.

Figure S15 QQ-plots from the EWAS meta-analysis of MZ versus DZ twins, highlighting methylation sites within previously published putative metastable epi-alleles.

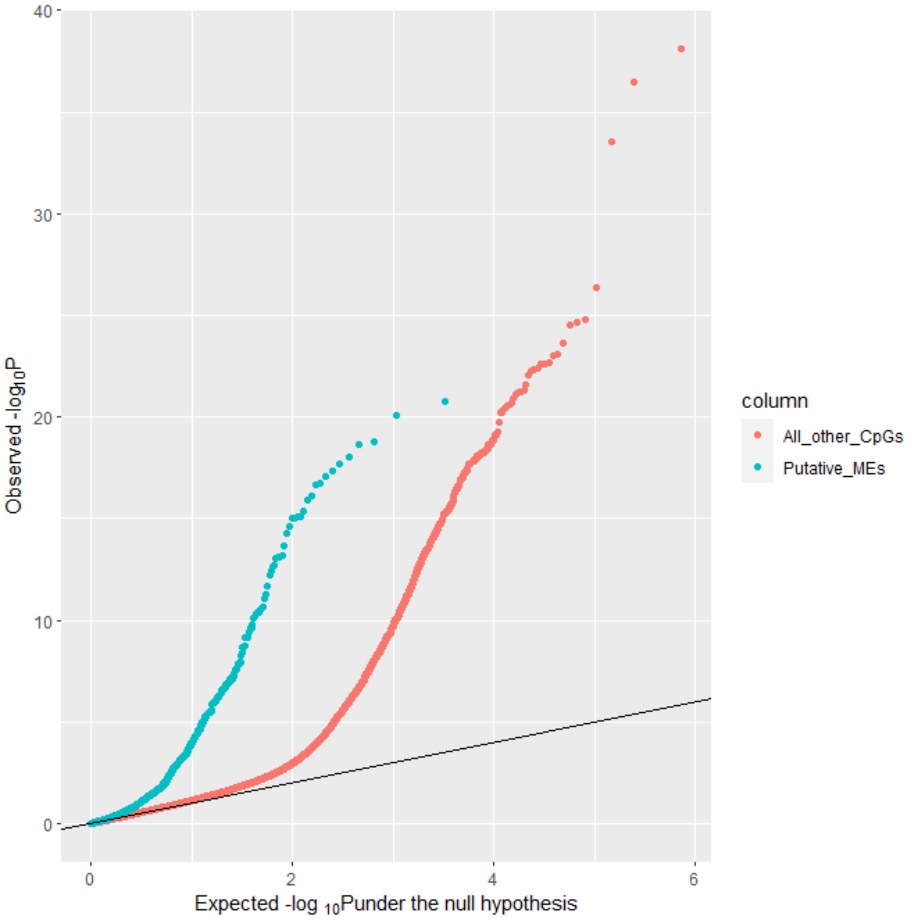

P-values from the EWAS meta-analysis (sample size = 5,723) are shown.

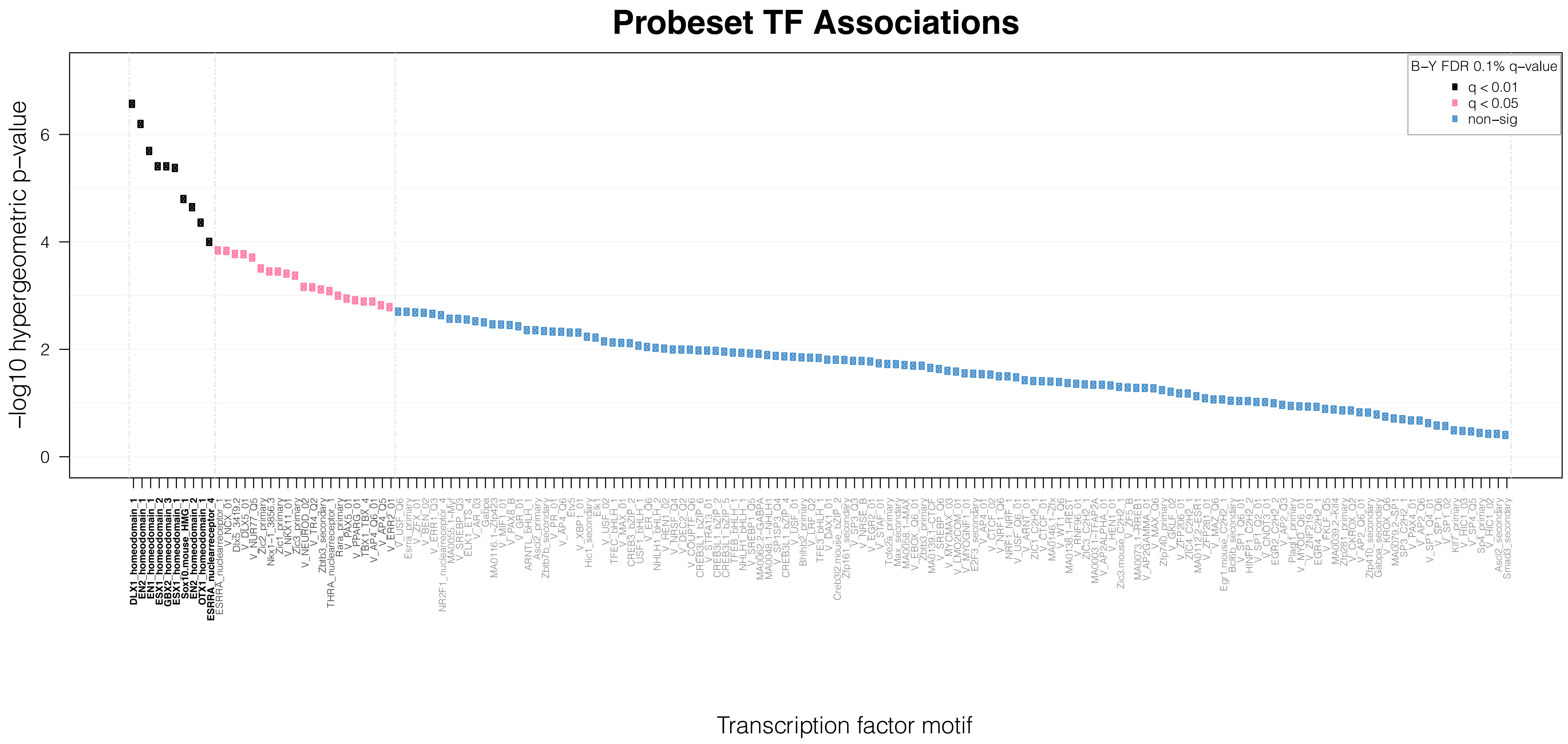
Probeset TF Associations
−log10 hypergeometric p−value
0
2
4
6
B−Y FDR 0.1% q−value
q < 0.01
q < 0.05
non−sig
Transcription factor motif

Figure S16 TF motif enrichment analysis of MZ-hypomethylated sites (previous page)

Results from the transcription factor (TF) motif enrichment analysis for MZ-hypomethylated sites. B-Y FDR= Benjamini–Yekutieli False Discovery Rate.

Figure S17 Top-enriched pathways of nearest genes of MZ-hypomethylated sites

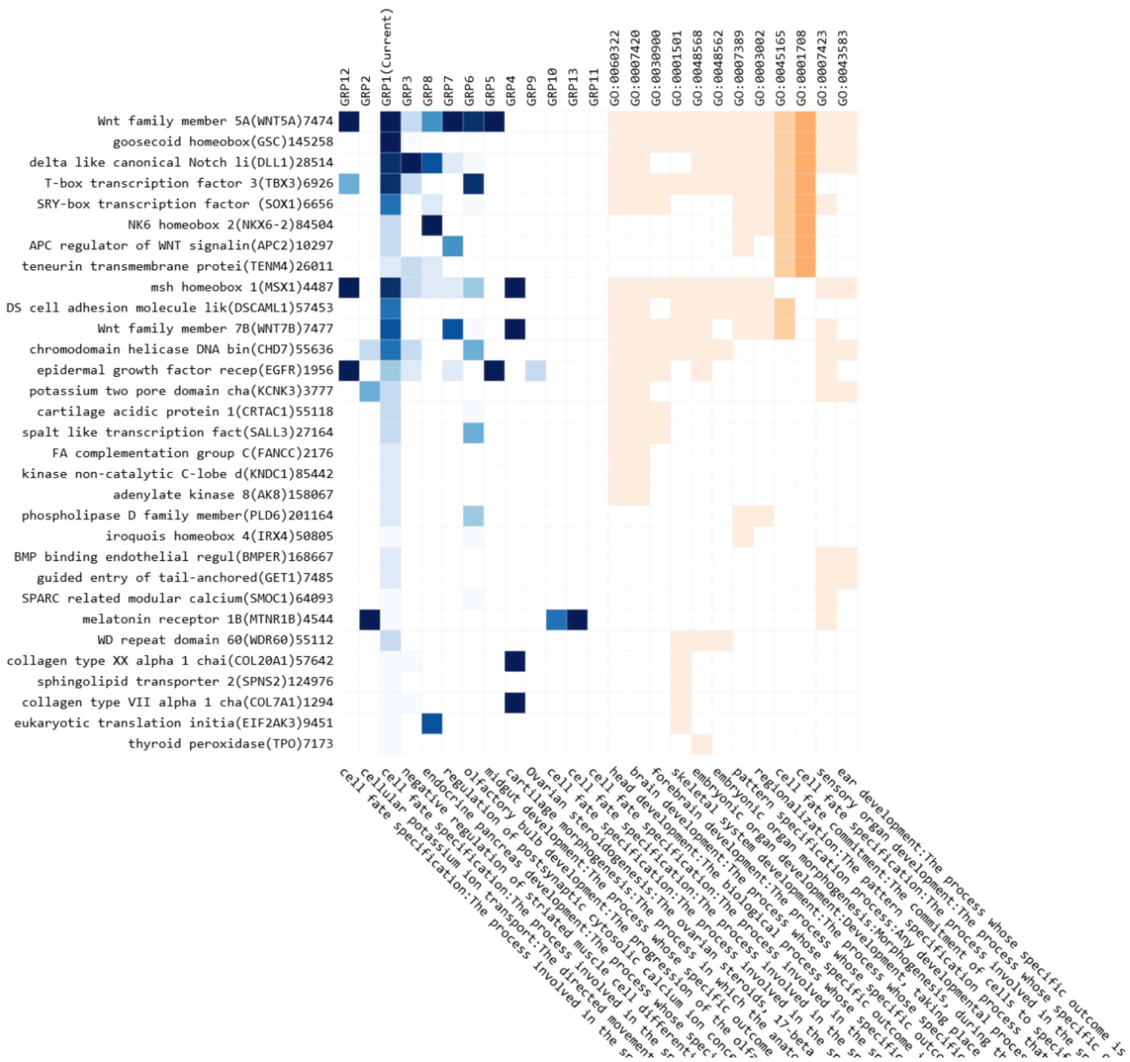

Clustergram showing the top-enriched pathways (cell fate; columns) for MZ-hypomethylated CpGs. Metascape automatically clusters similar GO terms (orange) into groups (GRP, blue). The clustergram shows the membership of genes (rows) involved in groups and GO-terms (columns). Each group consists of multiple GO terms, and each term consists of multiple genes. The rows show the genes that are implicated in the most strongly enriched group of pathways (GRP1; these pathways are related to cell fate; columns). A gene can be involved in many GO pathways of a particular group (dark blue) or can be involved in few (light blue) or no pathways (white) of a particular group. On the right, the orange heatmap shows genes across GO terms (within group 1). The darkness of the orange color reflect the p-value of the given term.

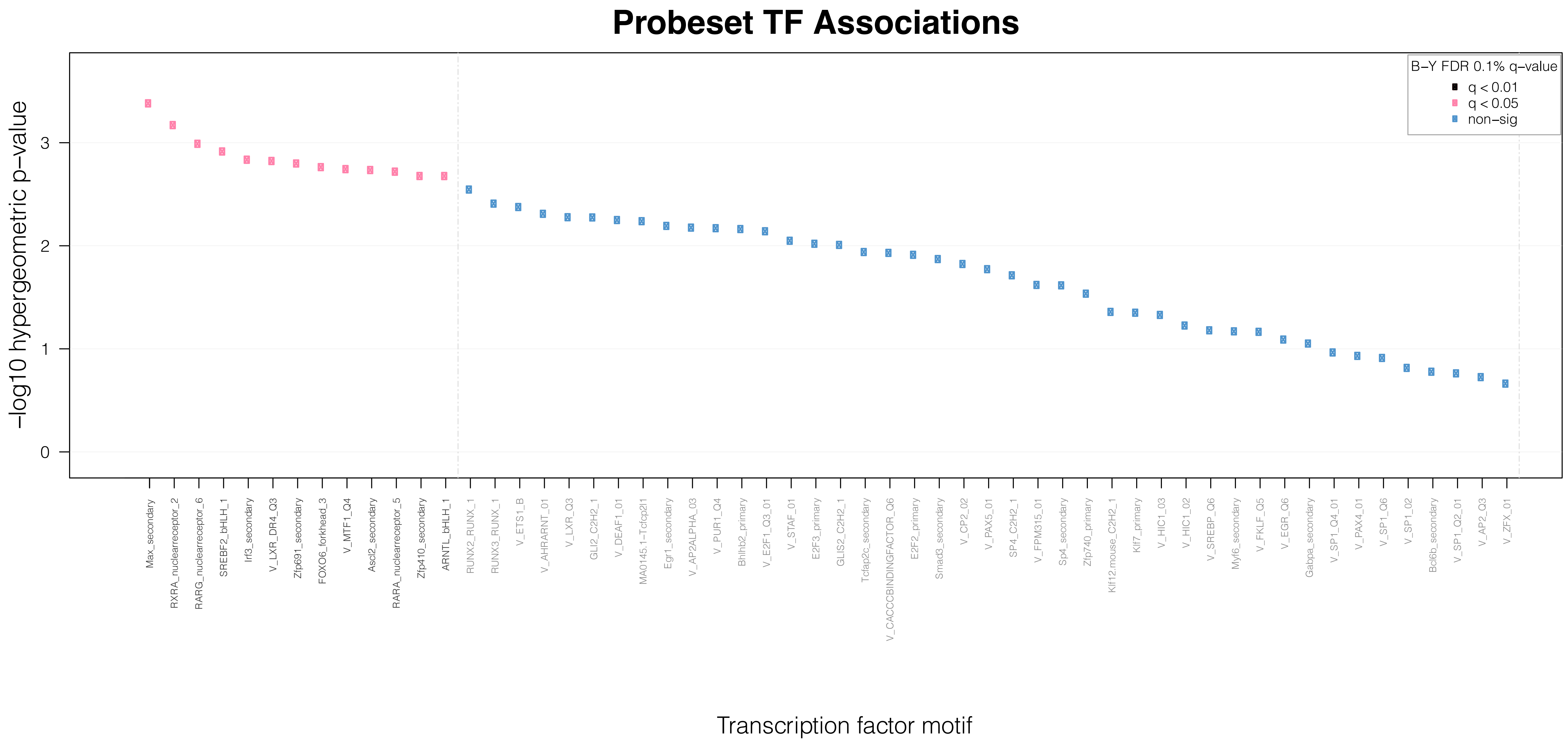

Probeset TF Associations
−log10 hypergeometric p−value
3
2
1
0
B−Y FDR 0.1% q−value
q < 0.01
q < 0.05
non−sig
Max_secondary
RXRA_nuclearreceptor_2
RARG_nuclearreceptor_6
SREBF2_bHLH_1
Irf3_secondary
V_LXR_DR4_Q3
Zfp691_secondary
FOXO6_forkhead_3
V_MTF1_Q4
Ascl2_secondary
RARA_nuclearreceptor_5
Zfp410_secondary
ARNTL_bHLH_1
RUNX2_RUNX_1
RUNX3_RUNX_1
V_ETS1_B
V_AHRARNT_01
V_LXR_Q3
GLI2_C2H2_1
V_DEAF1_01
MA0145.1-Tcfcp2l1
Egr1_secondary
V_AP2ALPHA_03
V_PUR1_Q4
Bhlhb2_primary
V_E2F1_Q3_01
V_STAF_01
E2F3_primary
GLIS2_C2H2_1
Tcfap2c_secondary
V_CACCCBINDINGFACTOR_Q6
E2F2_primary
Smad3_secondary
V_CP2_02
V_PAX5_01
SP4_C2H2_1
V_FPM315_01
Sp4_secondary
Zfp740_primary
Klf12.mouse_C2H2_1
Klf7_primary
V_HIC1_03
V_HIC1_02
V_SREBP_Q6
Myf6_secondary
V_FKLF_Q5
V_EGR_Q6
Gabpa_secondary
V_SP1_Q4_01
V_PAX4_01
V_SP1_Q6
V_SP1_02
Bcl6b_secondary
V_SP1_Q2_01
V_AP2_Q3
V_ZFX_01
Transcription factor motif

Figure S18 TF motif enrichment analysis of MZ-hypermethylated sites (previous page)

Results from the transcription factor (TF) motif enrichment analysis for MZ-hypermethylated sites. B-Y FDR= Benjamini–Yekutieli False Discovery Rate.

Figure S19 Top-enriched pathways of nearest genes of MZ-hypermethylated sites

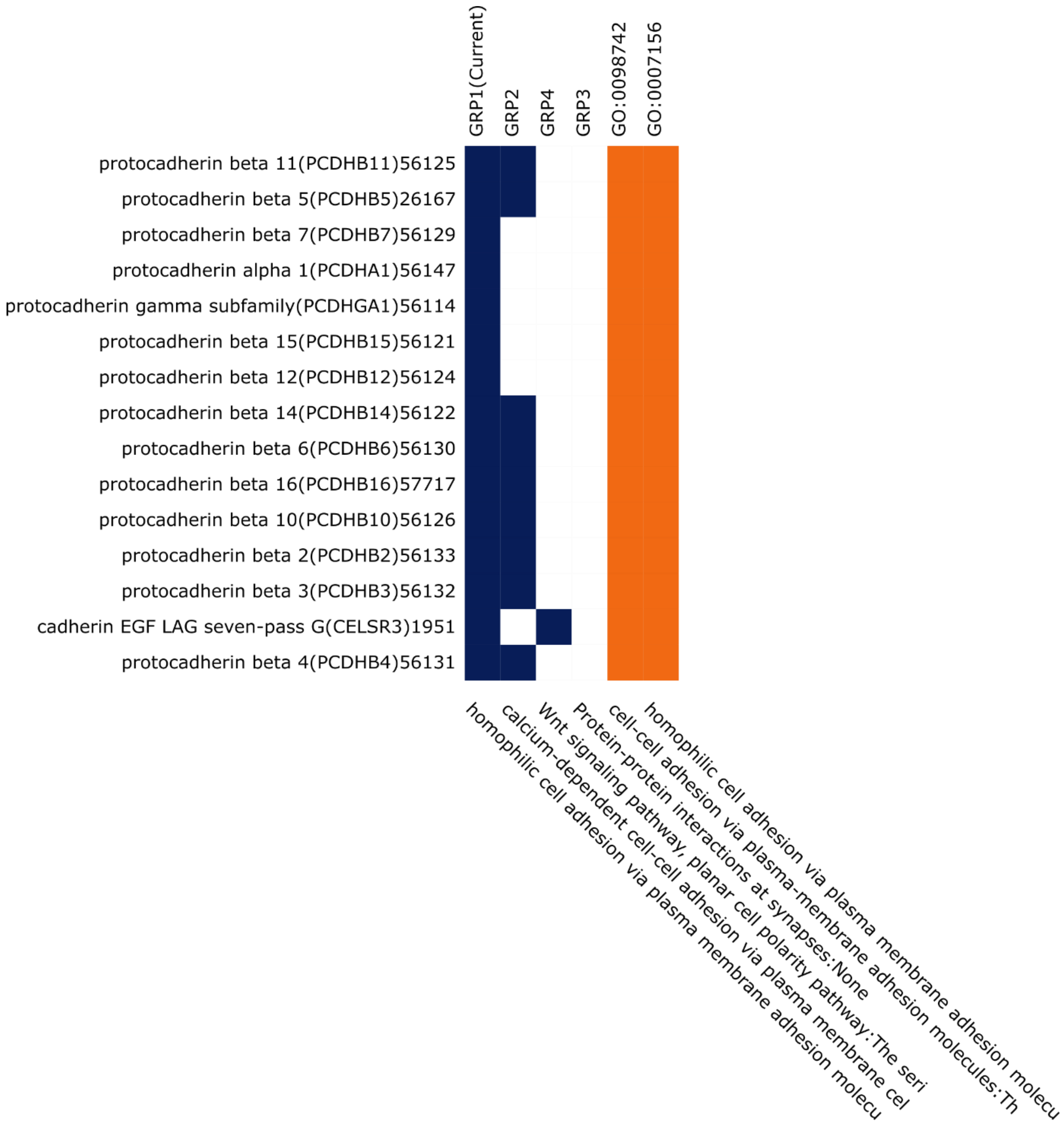

Clustergram showing the top-enriched pathways (cell adhesion; columns) for MZ-hypermethylated CpGs. Metascape automatically clusters similar GO-terms (orange) into groups (GRP, blue). The clustergram shows the membership of genes (rows) involved in groups and GO-terms (columns). Each group consists of multiple GO terms, and each term consists of multiple genes. The rows show the genes that are implicated in the most strongly enriched group of pathways (GRP1; these pathways are related to cell adhesion; columns). A gene can be involved in many GO pathways of a particular group (dark blue) or can be involved in few (light blue) or no pathways (white) of a particular group. On the right, the orange heatmap shows genes across GO terms (within group 1). The darkness of the orange color reflect the p-value of the given term.

Figure S20 MZ-DMPs are robust to correction for *cis* mQTLs.

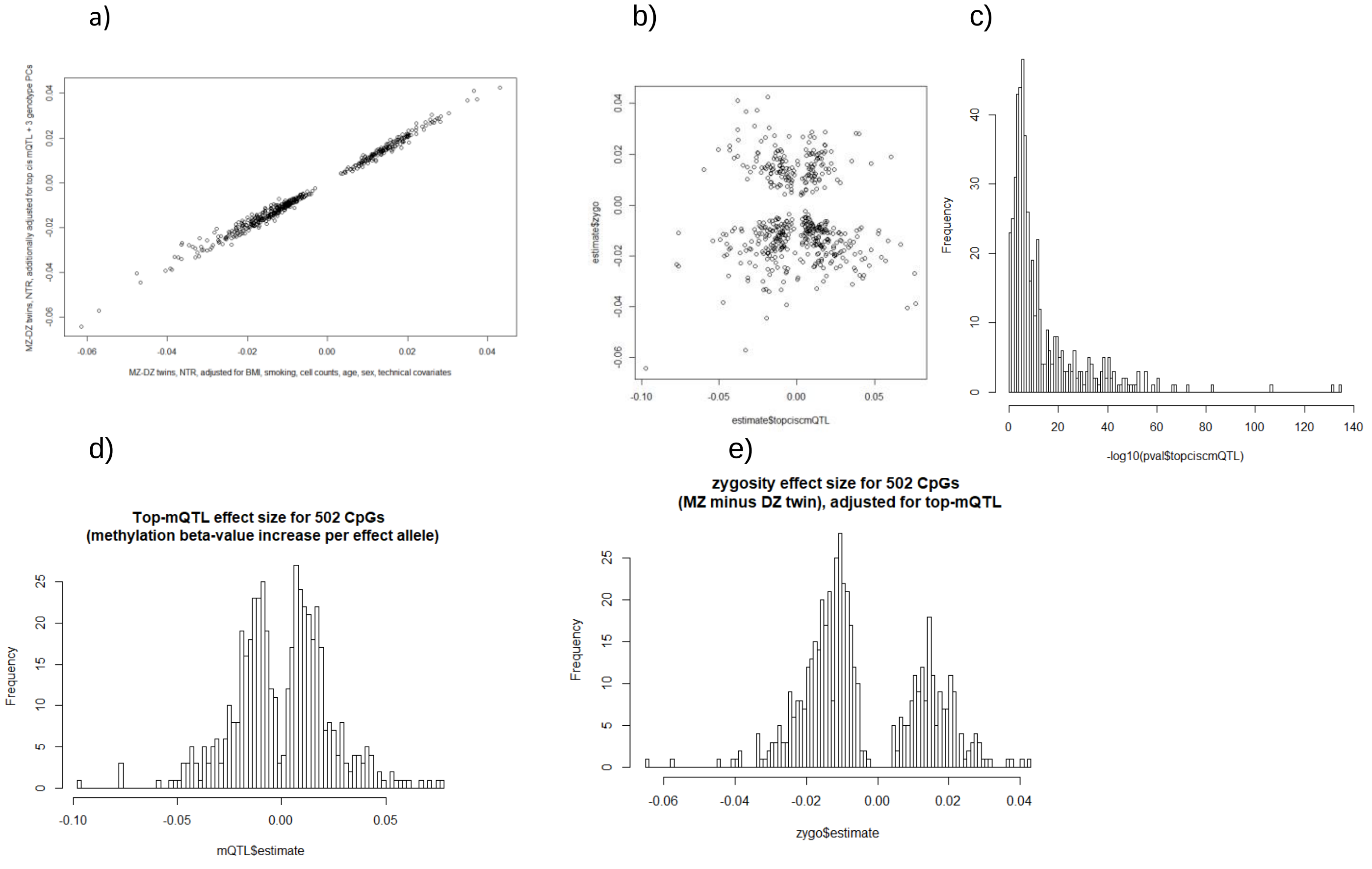

Results from the sensitivity analysis in NTR. a) Methylation difference between MZ and DZ twins from the primary EWAS in NTR (unadjusted for *cis* mQTL, x-axis; N=1957) versus methylation difference between MZ and DZ twins adjusting for the strongest *cis* mQTL for each CpG (y-axis, N=1713). b) Effect size of the strongest *cis* mQTL (x-axis) versus effect size of zygosity (methylation difference between MZ and DZ twins, y-axis). c) P-values for the *cis* mQTL effect confirming that these SNPs are strongly associated with methylation level in this sample. d) Effect sizes of *cis* mQTLs. e) Effect sizes for zygosity (MZ minus DZ twins), adjusted for the top *cis* mQTL.

Supplementary Tables

Supplementary Table 1 EWAS cohorts and Bayesian estimates of bias and inflation

**Whole blood**				
Cohort	Sample size	N methylation sites	Inflation	Bias
Brisbane SGS	613	367,620	1.03	0.0030
E-Risk	1,164	367,620	1.10	0.0103
Finland	1,708	367,620	1.80	-0.4163
NTR adults	1,957	367,620	1.03	0.2585
TwinsUK Study	492	367,620	0.88	-0.0161
Meta-analysis	5,723	367,620	1.03	-0.0344
**Buccal**				
Cohort	Sample size	N methylation sites	Inflation	Bias
NTR children	765	787,711	1.10	0.04

The R package Bacon was used to obtain Bayesian estimates of bias and inflation and to obtain bias- and inflation-corrected test statistics prior to meta-analysis. The estimates shown for the individual cohorts in this table represent the original estimates prior to adjustment. The meta-analysis estimates were obtained after adjusting the test statistics from the individual EWAS cohorts for bias and inflation with bacon and then meta-analysing the adjusted summary statistics.

Supplementary Table 2 Enrichment analysis results of telomeric and centromeric regions for MZ-hypomethylated sites

	all CpGs	Proportion	hypo-DMPs	Proportion	X-squared	df	p-value
centromere (5%)	22771	0.06	26	0.05	0.79393	1	0.3729
Telomeres (5%)	83164	0.23	224	0.45	143.27	1	< 2.2e-16
N total	367620		497				

Supplementary Table 3 Enrichment analysis results of telomeric and centromeric regions for MZ-hypermethylated sites

	all CpGs	Proportion	hyper-DMPs	Proportion	X-squared	df	p-value
centromere (5%)	22771	0.06	137	0.41	689.31	1	< 2.2e-16
Telomeres (5%)	83164	0.23	40	0.12	22.28	1	2.36E-06
N total	367620		337				

Supplementary Table 4 Enrichment analysis results of genomic regions for MZ-hypomethylated sites

Genomic Location	Odds Ratio	p Value	Input	Background
5'UTR	0	0.0173	0	0.009
1stExon	0.505	0.000393	0.05	0.095
TSS1500	0.327	9.77E-14	0.07	0.188
TSS200	0.457	2.71E-06	0.064	0.131
3'UTR	0	0.636	0	0.002
Intergenic	1.899	7.11E-11	0.34	0.214
Body	1.25	0.014	0.475	0.42
Island	3.361	1.41E-40	0.588	0.298
N_Shelf	0.252	1.97E-06	0.016	0.061
N_Shore	1.157	0.288	0.129	0.113
OpenSea	0.307	3.89E-26	0.149	0.363
S_Shelf	0.385	0.000215	0.024	0.06
S_Shore	0.892	0.51	0.095	0.105

Supplementary Table 5 Enrichment analysis results of genomic regions for MZ-hypermethylated sites

Genomic Location	Odds Ratio	p Value	Input	Background
5'UTR	0	8.29E-02	0	0.009
1stExon	2.597	7.24E-11	0.214	0.095
TSS1500	0.934	6.76E-01	0.178	0.188
TSS200	0.649	2.33E-02	0.089	0.131
3'UTR	0	1.00E+00	0	0.002
Intergenic	2.62	9.16E-17	0.415	0.214
Body	0.558	5.92E-07	0.288	0.42
Island	2.008	3.88E-10	0.46	0.298
N_Shelf	0.717	2.53E-01	0.045	0.061
N_Shore	2.32	2.40E-09	0.228	0.113
OpenSea	0.135	8.28E-36	0.071	0.363
S_Shelf	0.675	1.69E-01	0.042	0.06
S_Shore	1.558	5.57E-03	0.154	0.105

Supplementary Table 6 EWAS atlas enrichment analysis results for MZ-hypermethylated sites

Trait	Odds Ratio	p Value	DMC	Background
folic acid supplement during pregnancy	293.283	7.28E-154	39	470
neurodevelopmental presentations and congenital anomalies (ND/CAs)	65.216	1.21E-50	18	856
facial anomalies syndrome (ICF)	173.683	3.80E-37	10	181
Klinefelter syndrome	137.878	1.47E-28	8	179
thyroid lesion	18.729	2.25E-20	11	1753
Kabuki syndrome (KS)	17.342	1.09E-19	11	1891
follicular thyroid carcinoma	8.6	7.05E-19	16	5575
sperm viability	26.474	1.45E-17	8	898
Alzheimer's disease (AD)	19.204	3.58E-17	9	1392
preterm birth	4.986	7.87E-15	18	10662
fetal alcohol spectrum disorder (FASD)	31.094	2.26E-14	6	571
type 1 diabetes (T1D)	322.426	1.23E-13	3	30
gender	3.064	2.31E-09	21	15433
Claes-Jensen syndrome	13.957	6.21E-09	5	1050
leukoaraiosis (LA)	22.095	6.25E-09	4	531
primary Sjgren's Syndrome (pSS)	5.806	3.49E-07	7	3526
respiratory allergies (RA)	18.067	1.55E-06	3	485
maternal smoking	3.791	1.20E-05	8	6153
response to systemic corticosteroid	26.693	2.00E-05	2	219
maternal alcohol consumption	5.516	2.34E-05	5	2640
pediatric acute lymphoblastic leukemia	264.239	3.75E-05	1	12
osteoporosis	240.612	4.37E-05	1	13
pan-cancer	15.989	1.41E-04	2	364
human herpesvirus 6B infection	14.328	2.13E-04	2	406
maternal phthalate exposure	13.983	2.33E-04	2	416
lifetime estrogen exposure	70.56	4.28E-04	1	42
childhood stress	10.561	6.61E-04	2	550
ancestry	2.251	1.90E-03	9	10618
maternal pre-pregnancy body mass index (BMI)	26.499	2.81E-03	1	110
gestational diabetes mellitus	2.632	2.96E-03	6	6599
arsenic exposure	25.566	3.01E-03	1	114
urological cancer	22.732	3.76E-03	1	128
maternal pre-pregnancy obesity	18.047	5.86E-03	1	161
high-risk non-muscle invasive bladder cancer (HR-NMIBC)	14.152	9.29E-03	1	205

DMC=Differentially methylated CpGs - This is the number of methylation sites that is an MZ-DMP and has been previously associated with the trait reported in the first column.
Background= Total number of methylation sites previously associated with the trait in the first column.

Supplementary Table 7 EWAS atlas enrichment analysis results for MZ-hypomethylated sites

Trait	Odds Ratio	p Value	DMC	Background
Kabuki syndrome (KS)	69.641	9.46E-159	58	1891
ancestry	7.732	2.27E-42	42	10618
gender	5.357	1.56E-37	49	15433
breastfeeding	82.347	5.86E-31	10	252
respiratory allergies (RA)	46.22	1.20E-28	11	485
B Acute Lymphoblastic Leukemia with t(1;19)(q23;p13.3); E2A-PBX1 (TCF3-PBX1)	3.424	2.52E-21	44	21099
neurodevelopmental presentations and congenital anomalies (ND/CAs)	18.687	2.65E-15	8	856
ankylosing spondylitis	45.354	8.56E-14	5	222
maternal smoking	5.191	3.97E-13	16	6153
SETD1B-related syndrome	8.056	4.17E-13	11	2721
maternal pre-pregnancy body mass index (BMI)	74.224	6.08E-13	4	110
alcohol dependence	173.243	4.31E-12	3	37
maternal lead exposure	28.149	8.21E-12	5	355
follicular thyroid carcinoma	4.998	2.41E-11	14	5575
gestational diabetes mellitus	4.523	5.27E-11	15	6599
folic acid supplement during pregnancy	21.182	1.22E-10	5	470
plasma fasting HOMA-IR levels	561.24	3.62E-10	2	9
multiple sclerosis	3.928	3.91E-10	16	8093
colorectal laterally spreading tumor	3.855	6.13E-10	16	8245
household socioeconomic status in childhood	16.004	1.69E-09	5	620
intermittent explosive disorder	156.368	2.57E-08	2	27
sperm viability	11.02	5.31E-08	5	898
sedentary behavior	122.459	6.33E-08	2	34
multiple system atrophy	25.294	2.49E-05	2	157
high saturated fatty acids diet	5.009	5.03E-05	5	1965
cardiac autonomic responses (acceleration capacity)	216.014	5.74E-05	1	10
osteoporosis	163.521	9.48E-05	1	13
Alzheimer's disease (AD)	5.654	1.23E-04	4	1392
B Acute Lymphoblastic Leukemia with dic(9;20)(p11;q13)	4.132	2.34E-04	5	2379
thyroid lesion	4.483	5.63E-04	4	1753
perinatally-acquired HIV	4.498	2.63E-03	3	1309
childhood stress	7.144	2.71E-03	2	550
primary Sjgren's Syndrome (pSS)	2.779	4.25E-03	5	3526
maternal corticotropin-releasing hormone level	20.585	4.58E-03	1	96
response to antidepressants	14.169	9.30E-03	1	139

DMC=Differentially methylated CpGs - This is the number of methylation sites that is an MZ-DMP and has been previously associated with the trait reported in the first column.
Background= Total number of methylation sites previously associated with the trait in the first column.

Supplementary Table S8 Enrichment analysis results of age-VMPs for MZ-hypomethylated sites

	all CpGs	Proportion	hypo-DMPs	Proportion	X-squared	df	p-value
ageVMPs	5606	0.02	22	0.04	27.903	1	1.28E-07
N total	367620		497				

Supplementary Table S9 Enrichment analysis results of age-VMPs for MZ-hypermethylated sites

	all CpGs	Proportion	hyper-DMPs	Proportion	X-squared	df	p-value
ageVMPs	5606	0.02	35	0.10	176.36	1	< 2.2e-16
N total	367620		337				

Supplementary References

1. Chen, Y. *et al.* Discovery of cross-reactive probes and polymorphic CpGs in the Illumina Infinium HumanMethylation450 microarray. *Epigenetics* **2294,** (2013).

2. Zhou, W., Laird, P. W. & Shen, H. Comprehensive characterization, annotation and innovative use of Infinium DNA methylation BeadChip probes. *Nucleic Acids Res.* (2017). doi:10.1093/nar/gkw967

3. Hop, P. J. *et al.* Cross-reactive probes on Illumina DNA methylation arrays: a large study on ALS shows that a cautionary approach is warranted in interpreting epigenome-wide association studies. *NAR Genomics Bioinforma.* **2,** (2020).

4. Min, J. L. *et al.* Genomic and phenomic insights from an atlas of genetic effects on DNA methylation. *medRxiv* 2020.09.01.20180406 (2020). doi:10.1101/2020.09.01.20180406

5. Maldonado-Saldivia, J. *et al.* Dppa2 and Dppa4 Are Closely Linked SAP Motif Genes Restricted to Pluripotent Cells and the Germ Line . *Stem Cells* **25,** 19–28 (2007).

6. Masaki, H., Nishida, T., Kitajima, S., Asahina, K. & Teraoka, H. Developmental pluripotency-associated 4 (DPPA4) localized in active chromatin inhibits mouse embryonic stem cell differentiation into a primitive ectoderm lineage. *J. Biol. Chem.* **282,** 33034–33042 (2007).

7. Oliviero, G. *et al.* The variant Polycomb Repressor Complex 1 component PCGF1 interacts with a pluripotency sub-network that includes DPPA4, a regulator of embryogenesis. *Sci. Rep.* **5,** (2015).

8. Hernandez, C. *et al.* Dppa2/4 Facilitate Epigenetic Remodeling during Reprogramming to Pluripotency Cell Stem Cell Article Dppa2/4 Facilitate Epigenetic Remodeling during Reprogramming to Pluripotency. *Cell Stem Cell* **23,** 396–411 (2018).

9. Kessler, N. J., Waterland, R. A., Prentice, A. M. & Silver, M. J. Establishment of environmentally sensitive DNA methylation states in the very early human embryo. *Sci. Adv.* **4,** (2018).
