# Supplementary material for: Identical twins carry a persistent epigenetic signature of early genome programming
Source: Nat Commun. 2021 Sep 28;12:5618. doi: 10.1038/s41467-021-25583-7 (PMC8479069; doi:10.1038/s41467-021-25583-7)
Supplement: Supplementary file 3 — Description of Additional Supplementary Files [file 41467_2021_25583_MOESM3_ESM.pdf]

Description of Additional Supplementary Files

File Name: Supplementary Data 1
Description: Bonferroni significant CpGs detected in NTR and replication results in other twin cohorts

File Name: Supplementary Data 2
Description: Sensitivity analyses

File Name: Supplementary Data 3
Description: Genome-wide summary statistics from the EWAS meta-analysis

File Name: Supplementary Data 4
Description: Transcription factor binding motif enrichment analysis results for MZ-hypomethylated sites

File Name: Supplementary Data 5
Description: Pathway enrichment analysis results for transcription factors whose motifs are enrichmed among MZ-hypomethylated sites

File Name: Supplementary Data 6
Description: Pathway enrichment analysis results for nearest genes of MZ-hypomethylated sites

File Name: Supplementary Data 7
Description: Transcription factor binding motif enrichment analysis results for MZ-hypermethylated sites

File Name: Supplementary Data 8
Description: Pathway enrichment analysis results for transcription factors whose motifs are enrichmed among MZ-hypermethylated sites

File Name: Supplementary Data 9
Description: Pathway enrichment analysis results for nearest genes of MZ-hypermethylated sites

File Name: Supplementary Data 10
Description: mQTLs for MZ-hypomethylated sites

File Name: Supplementary Data 11
Description: mQTLs for MZ-hypermethylated sites

File Name: Supplementary Data 12
Description: Elastic net regression model results

File Name: Supplementary Data 13
Description: Weights from the elastic net regression model trained on meta-analysis significant probes to predict zygosity

File Name: Supplementary Data 14

Description: Weights from the elastic net regression model trained on meta-analysis significant probes to distinguish MZ twins from DZ twins and non-twins
