# Supplementary material for: Identical twins carry a persistent epigenetic signature of early genome programming
Source: Nat Commun. 2021 Sep 28;12:5618. doi: 10.1038/s41467-021-25583-7 (PMC8479069; doi:10.1038/s41467-021-25583-7)
Supplement: Supplementary file 11 — Reporting Summary [file 41467_2021_25583_MOESM11_ESM.pdf]

nature research

Corresponding author(s): Jenny van Dongen

Last updated by author(s): Jun 21, 2021

Reporting Summary

Nature Research wishes to improve the reproducibility of the work that we publish. This form provides structure for consistency and transparency in reporting. For further information on Nature Research policies, see our Editorial Policies and the Editorial Policy Checklist.

Statistics

For all statistical analyses, confirm that the following items are present in the figure legend, table legend, main text, or Methods section.

n/a	Confirmed	
☐	☒	The exact sample size (n) for each experimental group/condition, given as a discrete number and unit of measurement
☐	☒	A statement on whether measurements were taken from distinct samples or whether the same sample was measured repeatedly
☐	☒	The statistical test(s) used AND whether they are one- or two-sided<br>*Only common tests should be described solely by name; describe more complex techniques in the Methods section.*
☐	☒	A description of all covariates tested
☐	☒	A description of any assumptions or corrections, such as tests of normality and adjustment for multiple comparisons
☐	☒	A full description of the statistical parameters including central tendency (e.g. means) or other basic estimates (e.g. regression coefficient) AND variation (e.g. standard deviation) or associated estimates of uncertainty (e.g. confidence intervals)
☐	☒	For null hypothesis testing, the test statistic (e.g. F, t, r) with confidence intervals, effect sizes, degrees of freedom and P value noted<br>*Give P values as exact values whenever suitable.*
☒	☐	For Bayesian analysis, information on the choice of priors and Markov chain Monte Carlo settings
☒	☐	For hierarchical and complex designs, identification of the appropriate level for tests and full reporting of outcomes
☐	☒	Estimates of effect sizes (e.g. Cohen's d, Pearson's r), indicating how they were calculated

Our web collection on statistics for biologists contains articles on many of the points above.

Software and code

Policy information about availability of computer code

Data collection	NA

Data analysis	EWAS analyses were performed in R, with the R package gee.<br>DNA methylation data QC in individiual cohorts was performed with the following R packages: methylumi,wateRmelon, MethylAid, BMIQ, ENmix, RELIC, RCP, DNAmArray, meffil, omicsPrint.<br>Genotype data imputation was performed with mach-admix.<br>The sequence similarity of probes underlying significant MZ-DMPs detected in our meta-analysis was examined with the R package DNAmCrosshyb (https://github.com/pjhop/dnamarray_crossreactivity).<br>Meta-analysis was performed with METAL.<br>Enrichment analyses of previously reported traits and exposures, and genomic locations, were performed with the enrichment tool from the EWAS atlas.<br>To examine the overlap of differentially methylated sites with 15 Epigenomic Roadmap Chromatin States, we used eFORGE V2.0. Transcription factor (TF) motif analysis was performed using eFORGE-TF.<br>Pathway enrichment analysis on the nearest genes of significant DMPs was performed in metascape.<br>Penalized regression models were performed with the R package glmnet.<br>Imputation of missing values (DNA methylation data) was performed with the R package missMDA .<br><br>Code availability<br>An R-script (EpiPredictorMZtwin.R) and accompanying R data object to apply the epigenetic predictor of MZ twinning is provided in Supplementary Sofware 1. The pipeline for DNA methylation array analysis developed by the Biobank-based Integrative Omics Study (BIOS) consortium are available here: https://molepi.github.io/DNAmArray_workflow/ (DOI: 10.5281/zenodo.3355292). All other analysis code is available upon request from the corresponding author.

For manuscripts utilizing custom algorithms or software that are central to the research but not yet described in published literature, software must be made available to editors and reviewers. We strongly encourage code deposition in a community repository (e.g. GitHub). See the Nature Research guidelines for submitting code & software for further information.

Data

Policy information about availability of data

All manuscripts must include a data availability statement. This statement should provide the following information, where applicable:

- Accession codes, unique identifiers, or web links for publicly available datasets
- A list of figures that have associated raw data
- A description of any restrictions on data availability

Data availability

The HumanMethylation450 BeadChip data from the NTR are available as part of the Biobank-based Integrative Omics Studies (BIOS) Consortium in the European Genome-phenome Archive (EGA), under the accession code EGAD00010000887 [https://ega-archive.org/datasets/EGAD00010000887]. The HumanMethylation450 BeadChip data from E-Risk are accessible from the Gene Expression Omnibus (accession code: GSE105018; https://www.ncbi.nlm.nih.gov/geo/query/acc.cgi?acc=GSE105018). The FTC DNA methylation data will be deposited in THL Biobank Finland, from which researchers will be able to apply for access. The majority of MZ TwinsUK whole blood DNA methylation profiles are a subset of publicly available dataset GEO GSE121633 (https://www.ncbi.nlm.nih.gov/geo/query/acc.cgi?acc=GSE121633). Additional individual-level data are not permitted to be shared or deposited due to the original consent given at the time of data collection. However, access to these data can be applied for through the TwinsUK data access committee. For information on access and how to apply, see http://www.twinsuk.ac.uk/data-access/submission-procedure-2/. BSGS DNA methylation data are available at the Gene Expression Omnibus under accession code GSE56105 (https://www.ncbi.nlm.nih.gov/geo/query/acc.cgi?acc=GSE56105). The NTR-ACTION datasets are available from the Netherlands Twin Register on reasonable request (https://tweelingenregister.vu.nl/information_for_researchers/working-with-ntr-data).

Genome-wide summary statistics from the EWAS meta-analysis and weights from the elastic net regression models are provided in Supplementary Data 3, Supplementary Data 13, and Supplementary Data 14.

Field-specific reporting

Please select the one below that is the best fit for your research. If you are not sure, read the appropriate sections before making your selection.

[x] Life sciences [ ] Behavioural & social sciences [ ] Ecological, evolutionary & environmental sciences

For a reference copy of the document with all sections, see nature.com/documents/nr-reporting-summary-flat.pdf

Life sciences study design

All studies must disclose on these points even when the disclosure is negative.

Sample size	We performed a meta-analysis of all existing DNA methylation data that was available to us (and had been collected previously for other purposes).
Data exclusions	Bad quality samples were excluded, as described in the methods section
Replication	A discovery EWAS was performed followed by replication. Replication analysis in four independent twin cohorts revealed strong concordance of effects (Figs 1a-d, Supplementary Data 1): correlations of effect sizes ranged from 0.84 to 0.97. The number of DMPs that replicated following Bonferroni correction for 243 tests ranged from 5 to 186. Since effect sizes were very similar across cohorts (Figs 1a-d), differences between cohorts in the number of DMPs that replicated following stringent Bonferroni correction likely reflect power related to the following differences between replication cohorts: total sample size (ranging from 356-1708), zygosity frequencies (ranging from 33% to 80% MZ), and whether correction for inflation of test statistics was required (Table S1). DMPs identified in blood samples from adult twins also showed

	strong concordance of effects in buccal samples from children (Fig. 1e; r=0.87).<br><br>Next a meta-analysis of all datasets was performed.
Randomization	Not applicable, this is not an experimental (i.e. intervention) study.
Blinding	Not applicable, this is not an experimental (i.e. intervention) study.

Reporting for specific materials, systems and methods

We require information from authors about some types of materials, experimental systems and methods used in many studies. Here, indicate whether each material, system or method listed is relevant to your study. If you are not sure if a list item applies to your research, read the appropriate section before selecting a response.

Materials & experimental systems

n/a	Involved in the study
☒	☐ Antibodies
☒	☐ Eukaryotic cell lines
☒	☐ Palaeontology and archaeology
☒	☐ Animals and other organisms
☐	☒ Human research participants
☒	☐ Clinical data
☒	☐ Dual use research of concern

Methods

n/a	Involved in the study
☒	☐ ChIP-seq
☒	☐ Flow cytometry
☒	☐ MRI-based neuroimaging

Human research participants

Policy information about studies involving human research participants

Population characteristics	Monozygotic and dizygotic twins from multiple twin registers (for each cohort, descriptives are provided in table 1 of the manuscript).
Recruitment	Twins (monozygotic; MZ and dizygotic; DZ) participate on a voluntary basis in ongoing longitudinal studies (details are desribed for each twin cohort in the methods section). MZ twins are a bit more eager to participate in research compared to DZ twins, resulting in larger overall numbers of registred MZ twins in twin registers, however, this does not influence the current study results, because methylation array data were generated on sub-sets of participants from each twin register (both MZ and DZ twins). Furthermore, smaller numbers of DZ twins would only lead to lower power (not bias), and our results indicate that our study was highly powered to detect methylation differences between MZ and DZ twins.
Ethics oversight	This is indicated for each of the respective twin cohorts in the participants and samples section in the methods. The protocol for each study was approved by the ethical review board of each institution. Informed consent was obtained for all participants.

Note that full information on the approval of the study protocol must also be provided in the manuscript.
